# Hologenomics Reveals Specialized Dietary Adaptations in the Mengla Snail‐Eating Snake

**DOI:** 10.1002/advs.202509999

**Published:** 2025-07-16

**Authors:** Chaochao Yan, Xin‐Ning Li, Zhong‐Liang Peng, Wei Wu, Zeng Wang, Zhao‐Ran Zhu, Jia‐Chang Liu, Yao Wang, Jin‐Long Ren, Zhi‐Yi Zhang, Jia‐Tang Li

**Affiliations:** ^1^ Mountain Ecological Restoration and Biodiversity Conservation Key Laboratory of Sichuan Province, Chengdu Institute of Biology Chinese Academy of Sciences Chengdu 610213 China; ^2^ China‐Croatia Belt and Road Joint Laboratory on Biodiversity and Ecosystem Services, Chengdu Institute of Biology Chinese Academy of Sciences Chengdu 610213 China; ^3^ University of Chinese Academy of Sciences Beijing 100049 China; ^4^ Southeast Asia Biodiversity Research Institute Chinese Academy of Sciences Yezin Nay Pyi Taw 05282 Myanmar

**Keywords:** adaptive evolution, genomics, hologenome, metagenomics

## Abstract

Serpents, as highly adaptable vertebrates, provide robust models for studying the mechanisms of dietary specialization. Using an integrative multi‐omics approach, encompassing host genomic, transcriptomic, proteomic, gut metagenomic, and enzymatic analyses, the mechanisms underlying dietary adaptations in the Mengla snail‐eating snake (*Pareas menglaensis*), a species specialized in consuming snails is investigated. Adaptations supporting this diet included evolution of infralabial glands secreting toxin homologs and digestive enzymes, facilitating molluscan predation and digestion. This specialization has driven adaptive evolution in the host genome and shaped the gut microbiota, addressing both nutritional challenges (e.g., lipid deficiency) and digestive requirements (e.g., mucus degradation) associated with snail consumption. Notably, the functional convergence in microbial gene functions between reptiles and mammals highlights parallel evolutionary pathways in dietary specialization. These findings elucidate the genomic foundations of dietary specialization in *P. menglaensis*, offering broader insights into evolutionary adaptation within a holobiome framework.

## Graphical abstract

Dietary adaptation studies from a holobiome perspective are scarce beyond mammals. This research reveals how genomic and microbial interactions drive dietary specialization in the Mengla snail‐eating snake, with evidence of gut symbiont convergence between reptiles and mammals. These findings broaden the understanding of cross‐species dietary adaptations and emphasize the hologenome's key role in evolution.

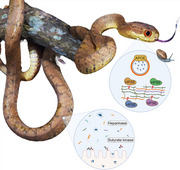

## 1 Introduction

**Figure 1:**
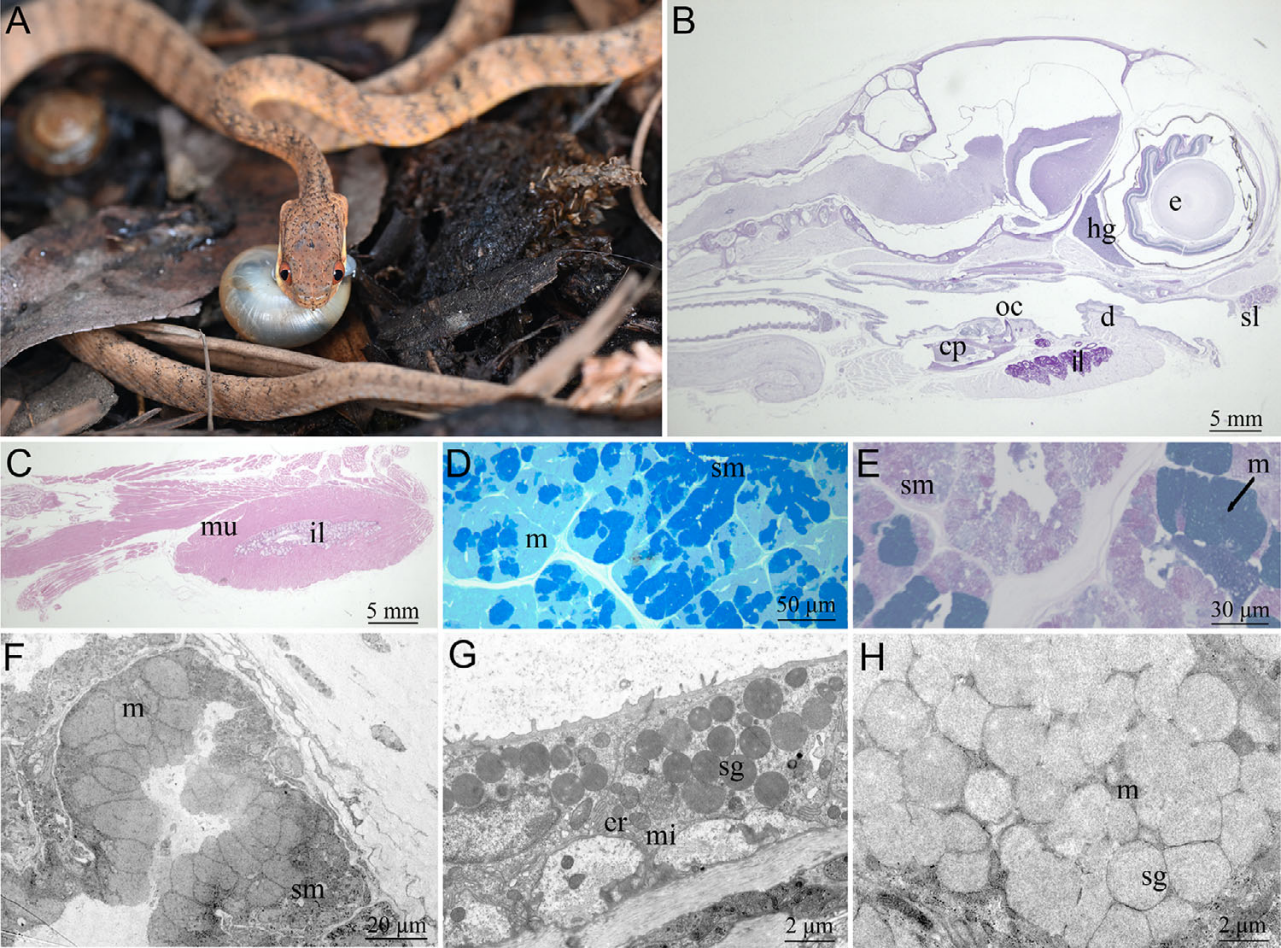
Histomorphology of specialized infralabial gland in *Pareas menglaensis*. A) *Pareas menglaensis* preying on a snail. B) Parasagittal plane of head stained with hematoxylin & eosin, showing location of hypertrophied infralabial gland. Scale bar = 5 mm. C) Parasagittal plane of mandible, showing entire infralabial gland wrapped by muscle. Scale bar = 5 mm. D) Enlarged tissue section of infralabial gland, showing seromucous cells with a stronger positive response to Coomassie brilliant blue R250. E) Infralabial gland, showing seromucous cells with a positive response to periodic acid‐Schiff (PAS) and mucous cells with a positive response to Alcian blue, pH 2.5. Scale bar = 30 µm. F) Ultrastructure of acinus in the infralabial gland, revealing cells with secretory granules of different electron densities. Scale bar = 20 µm. G) Seromucous cells with highly electron‐dense, variably‐sized secretory granules inside. Scale bar = 2 µm. H) Mucous cells with homogeneous, moderately electron‐dense secretory granules inside. Scale bar = 2 µm. e, eye; hg, Harderian gland; oc, oral cavity; sl, supralabial gland; cp, compound bone; il, infralabial gland; d, dentary bone; mu, muscle; sm, seromucous cells; m, mucous cells; sg, secretory granules; mi, mitochondria; er, endoplasmic reticulum.

Dietary shifts have profoundly influenced species radiation into new ecological niches, driving both physiological and morphological adaptations.^[^ ^1^, ^2^ ^]^ Although research on dietary specialization and its genetic underpinnings has primarily focused on mammals,^[^ ^1^, ^2^, ^3^ ^]^ serpents also exhibit considerable dietary adaptability, with diverse strategies ranging from polyphagy to oligophagy, including specializations in crustacean‐eating, egg‐eating, and ant‐eating.^[^ ^4^, ^5^, ^6^, ^7^ ^]^ Within this context, *Pareas*, a unique snake lineage that feeds exclusively on terrestrial mollusks, such as snails and slugs (**Figure** **1A**), serves as an ideal model for studying the genetic mechanisms underlying dietary specialization.

Long‐term adaptations to specialized diets can induce distinct genetic modifications within organisms. For instance, the low‐nutrient, high‐fiber bamboo diet of the giant panda (*Ailuropoda melanoleuca*) has driven the pseudogenization of the umami taste receptor gene *TAS1R1* and positive selection of protein digestion‐related genes, such as *PRSS1*, *PRSS36*, and *CPB1*, thus enhancing digestion and nutrient absorption efficiency.^[^ ^2^ ^]^ Similarly, the loss of certain genes, such as *ERN2*, *CTRL*, and *REP15*, in vampire bats has been linked to adaptations for blood feeding, addressing nutrient composition biases inherent to hematophagy.^[^ ^3^ ^]^ Likewise, mollusk‐exclusive feeding presents two primary challenges for pareid snakes. First, snails secrete substantial amounts of glycosaminoglycan‐rich mucus (e.g., heparan sulfate/heparin, dermatan sulfate, and chondroitin sulfate) when threatened, which can obstruct the trachea and impede respiration.^[^ ^8^ ^]^ Second, the nutrient profile of snails, particularly low lipid content,^[^ ^9^ ^]^ complicates energy storage and cellular function. Recent studies suggest that certain *Pareas* species have evolved hypertrophied infralabial glands in their lower jaw that produce secretions facilitating mollusk digestion, representing a unique adaptation to their specialized diet.^[^ ^10^ ^]^ While morphological adaptations associated with snail consumption in *Pareas* have been minimally documented, the genetic basis for this dietary specialization remains largely unexplored.

Dietary specializations are frequently linked to changes in host‐associated microbiomes, where interactions between host genomes and microbiomes (i.e., “hologenomes”) play an integral role in these adaptations.^[^ ^1^, ^11^ ^]^ The vampire bat exemplifies co‐evolutionary adaptation, having specialized to consume a blood‐based diet, characterized by high protein content, low carbohydrates, and inherent pathogen exposure. This dietary shift drove substantial changes in both its genome and gut microbiome,^[^ ^11^ ^]^ with considerable gastrointestinal tract enrichment with commensals containing urea metabolism‐related genes (e.g., *ureA*) and potentially protective bacteria (e.g., *Amycolatopsis*), enhancing nitrogen metabolism and pathogen resistance.^[^ ^11^ ^]^ Similarly, the Malayan pangolin has developed specific dietary adaptations to an exclusive ant‐ and termite‐based diet, which is high in chitin.^[^ ^1^ ^]^ This adaptation is marked by the up‐regulation of the acidic mammalian chitinase gene, facilitating efficient chitin breakdown,^[^ ^12^ ^]^ along with gut microbiome enrichment with microorganisms expressing *TREH*, which aids in the digestion of trehalose, a product of chitin degradation.^[^ ^1^ ^]^ However, whether specific adaptations in gut microbiomes accompany genetic changes in pareid snakes, and how the hologenome supports dietary specialization, have not yet been clarified.

This study employed an integrative multi‐omics approach, combining host genomic, transcriptomic, proteomic, and gut metagenomic data, alongside assays validating key enzyme activities, to elucidate the hologenomic mechanisms underlying dietary specialization in the Mengla snail‐eating snake (*Pareas menglaensis*). Analysis revealed various genes under species‐specific selection, including genes involved in glycosaminoglycan and lipid metabolism, which facilitate snail digestion and nutrient utilization. Additionally, complex and diverse interactions between the host and its symbiotic microbiota were identified, highlighting adaptive responses to mucus digestion and the nutritional constraints of a low‐lipid diet. Overall, this study advances our understanding of the genetic foundations of dietary specialization and provides novel insights into the coordination of physiological functions through host‐symbiont interactions.

## 2 Results

### 2.1 Histomorphology of Specialized Infralabial Gland in *P. Menglaensis*

Tissue analysis revealed a hypertrophied infralabial gland in the lower jaw of *P. menglaensis* (Figure 1A). The gland, extending from the anterior tip of the dentary to the front of the compound bone, was attached to the mandible and encased in muscle tissue (Figure 1B,C). The gland was primarily composed of mucous cells (m) and seromucous cells (sm), organized into acinar lobules of varying sizes (Figure 1D,E). Seromucous cells, in contrast to mucous cells, demonstrated positive staining with PAS and Coomassie Brilliant Blue R250, whereas mucous cells showed a marked positive reaction to Alcian blue at pH 2.5, indicating differential cytoplasmic composition between the two cell types (Figure 1D,E).

Ultrastructural analysis provides precise insights into the subcellular architecture of secretory cells, where elevated electron density within secretory granules reflects high protein content.^[^ ^13^, ^14^ ^]^ Examination of the infralabial gland in *P. menglaensis* revealed that acinar cells contained secretory granules exhibiting varying electron densities (Figure 1F). Seromucous cells displayed highly electron‐dense, variably‐sized secretory granules (Figure 1G) and a cytoplasm rich in organelles associated with protein synthesis and secretion, including mitochondria and rough endoplasmic reticulum (Figure 1G). In contrast, mucous cells were characterized by homogeneous, uniformly sized, moderately electron‐dense secretory granules (Figure 1H).

### 2.2 Dietary Adaptations cause Genetic Changes in *P.Menglaensis*

**Figure 2:**
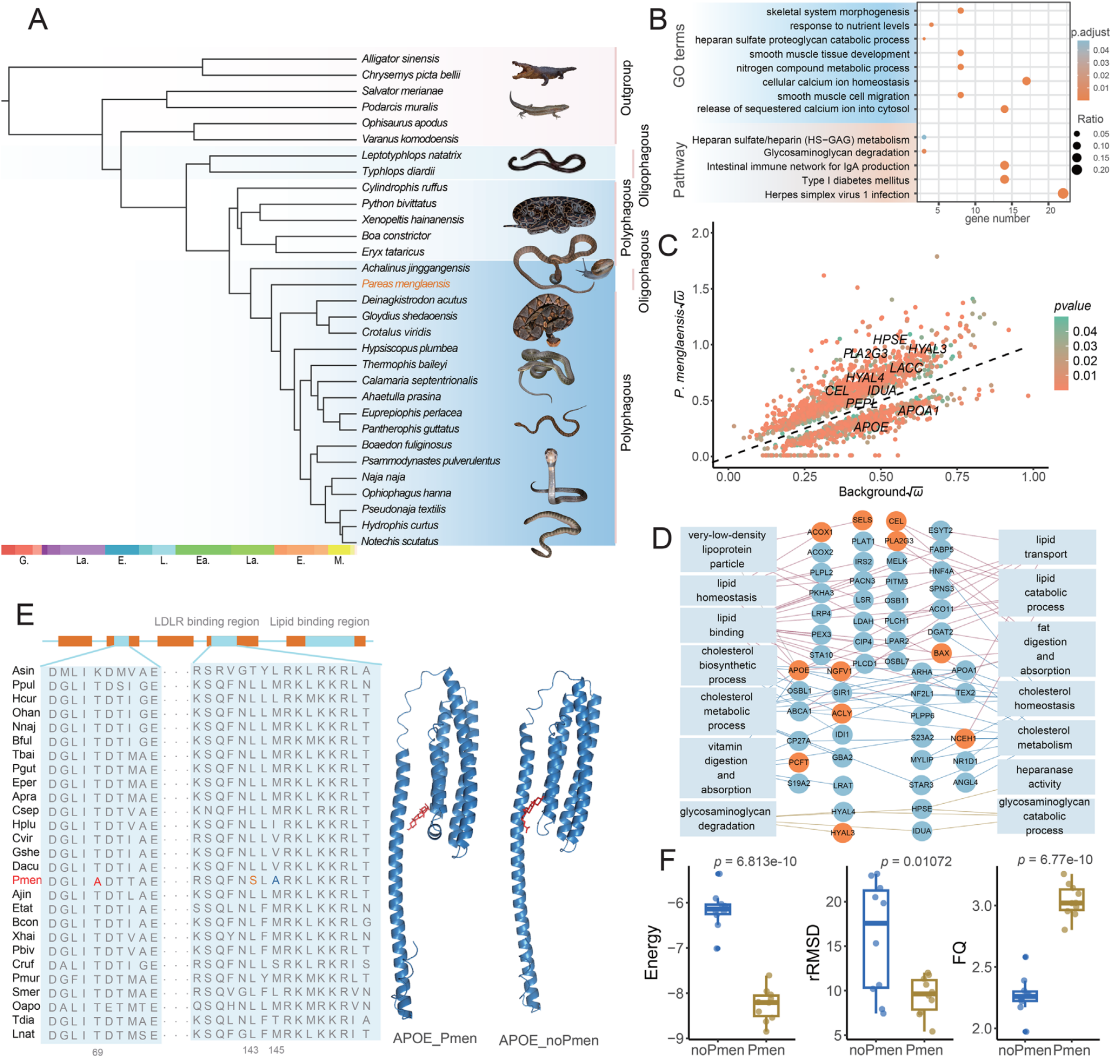
Comparative genomic analysis of *Pareas menglaensis* and non‐snail‐eating reptiles. A) Phylogenetic tree of 31 species analyzed in this study, with dietary classifications (oligophagy or polyphagy) labeled on the right. Timescale bar at the bottom shows divergence time. B) Significantly enriched Gene Ontology (GO) terms (blue) and pathways (red) associated with expanded genes. Bubble size represents the number of genes in a certain category divided by the total number of annotations for the corresponding term. Color indicates adjusted *p*‐values. C) Scatter plot of ω values for *P. menglaensis* genes compared to background genes, highlighting genes with accelerated molecular evolution (i.e., rapidly evolving genes, REGs) in *P. menglaensis*. Color indicates the significance of differences in evolutionary rates. D) Functional enrichment analysis of REGs, represented by filled blue circles, with REGs under positive selection shown as filled orange circles. Colored lines depict relationships between genes and their metabolic functions: red for lipid metabolism, blue for cholesterol metabolism, yellow for glycosaminoglycan degradation and absorption. E) Sequence alignment showing three *P. menglaensis*‐specific amino acid substitutions (in different colors) in APOE. Orange bars indicate α‐helix of APOE, blue bars indicate structural‐functional domains. 3D models of APOEs from *P. menglaensis* (Pmen) and other snakes (noPmen) docking with cholesterol are displayed on the right. F) Boxplots showing differences in binding models between APOEs from *P. menglaensis* and other snakes, with each model subjected to 10 docking simulations. *P*‐values were calculated using two‐sided Student's *t*‐test and are presented above each plot. RMSD, root mean square deviation; FQ, fit quality. The whiskers indicate 1.5 times of the interquartile range.

A comparative analysis involving genomic data from 31 reptile species, including *P. menglaensis*, was conducted to investigate genetic changes associated with its specialized snail‐eating behavior (**Figure** **2**A; Figure S1, Supporting Information). Genomic data for *P. menglaensis* were obtained from Peng et al. (2023)^[^ ^15^ ^]^ and Wang et al. (2023)^[^ ^16^ ^]^ (Table S1, Supporting Information). This analysis identified 64 gene families with significant expansions unique to *P. menglaensis* (Table S2, Supporting Information). Functional enrichment analysis revealed that the most significantly enriched functions (i.e., lowest *p*‐values) among the expanded genes were associated with immune responses, particularly against infections of orofacial mucosal surfaces, such as herpes simplex virus 1, and with the modulation of cellular calcium ion levels. These genes were also significantly enriched in pathways related to glycosaminoglycan degradation, especially heparan sulfate/heparin metabolism (R‐MMU‐1638091), in which heparanase (HPSE) plays a central role. Furthermore, notable gene family expansions were detected in categories related to nutrient sensing (e.g., INS) and skeletal system morphogenesis (GO:0031667 and GO:0048705) (Figure 2B, Table S3, Supporting Information). In contrast, contractions were identified in gene families associated with key physiological functions, including TRIM family members involved in hepatic lipid metabolism and insulin sensitivity,^[^ ^17^, ^18^ ^]^ olfactory receptor genes linked to fatty acid oxidation and hormone‐sensitive lipase activation,^[^ ^19^, ^20^ ^]^ and some other immune‐related genes (Table S4, Supporting Information).

In *P. menglaensis*, genomic analyses identified 1846 rapidly evolving genes (REGs; Figure 2C and Table S5, Supporting Information) and 963 positively selected genes (PSGs; Table S6, Supporting Information), reflecting widespread signatures of adaptive evolution. These genes were significantly enriched in glycosaminoglycan metabolism (e.g., GO:0015012, GO:0030206, GO:0030201, and hsa00531) and lipid metabolism (e.g., GO:0006629, GO:0033540, hsa00062, and hsa01212) (Figure 2D, Tables S7 and S8, Supporting Information), and included *HPSE*, *IDUA*, and *HYAL4*, which encode enzymes that hydrolyze heparan sulfate/heparin, dermatan sulfate, and chondroitin sulfate, respectively.^[^ ^21^, ^22^, ^23^ ^]^ Genes involved in lipid transport and processing, such as *APOE*, *APOA1*, *PLA2G3*, and *CEL*, also exhibited signatures of positive selection and/or accelerated evolution (Figure 2D). To validate these findings, an independent analysis was performed using the HyPhy platform. Consistently, *APOE*, *PLA2G3*, and *CEL* were again identified as PSGs (Table S9, Supporting Information) and *APOE* was again identified as a REG (Table S10, Supporting Information). *HPSE*, *IDUA*, and *HYAL4* were also recovered as REGs in both analyses (Table S10, Supporting Information). HyPhy further revealed significant enrichment of PSGs in glycosaminoglycan metabolism (e.g., GO:0005539 and hsa00531) and lipid metabolism (e.g., GO:0033540 and hsa00062) (Table S11, Supporting Information), along with REGs enriched in lipid‐related functions (e.g., GO:0019216 and GO:0042159) (Table S12, Supporting Information).

Among the genes under natural selection in *P. menglaensis*, 1623 carried species‐specific amino acid substitutions, many of which were involved in lipid metabolism (Table S13, Supporting Information). Notably, APOE, a lipid metabolism‐related gene identified as both a PSG and REG, harbored *P. menglaensis*‐specific mutations (Table S14, Supporting Information). Given its critical role in coordinating lipid metabolism through binding triglycerides, glycosaminoglycans, and low‐density lipoprotein receptors (LDLRs), these mutations may contribute substantially to fat metabolism adaptations in this species.

Three *P. menglaensis*‐specific amino acid substitutions were identified in *APOE* across the species comparison. The threonine‐to‐alanine substitution at amino acid position 69 (T69A) was predicted to impact protein structure (PROVEAN score < −2.5), while two additional substitutions, L143S and V145A, were located within the LDLR‐binding domain, a functionally important region of the protein (Figure 2E; Figure S2, Supporting Information). To evaluate the functional impact of these substitutions, molecular docking simulations were performed to compare lipid‐binding affinity between APOE variants from *P. menglaensis* and other snakes. Notably, the *P. menglaensis* variant demonstrated markedly enhanced binding characteristics for lipids such as cholesterol, including increased stability and higher affinity (Figure 2F, Table S15, Supporting Information). These enhancements were supported by reduced binding free energy and improved fit quality (FQ). Consistent results were obtained using AlphaFold3, a platform for biomolecular interaction prediction, which indicated superior lipid‐binding stability for *P. menglaensis* APOE based on significantly elevated ptm and iptm model confidence scores (Figure S3, Supporting Information). To further resolve receptor interactions, AlphaFold3 was employed to model APOE‐LDLR interfaces. Although no significant difference in overall LDLR‐binding capacity was detected between *P. menglaensis* and other snakes, the optimal conformation of the *P. menglaensis* APOE‐LDLR complex displayed enhanced structural stability (Figure S3, Supporting Information).

### 2.3 Infralabial Gland‐Specific Gene Expression in *P. Menglaensis*

**Figure 3:**
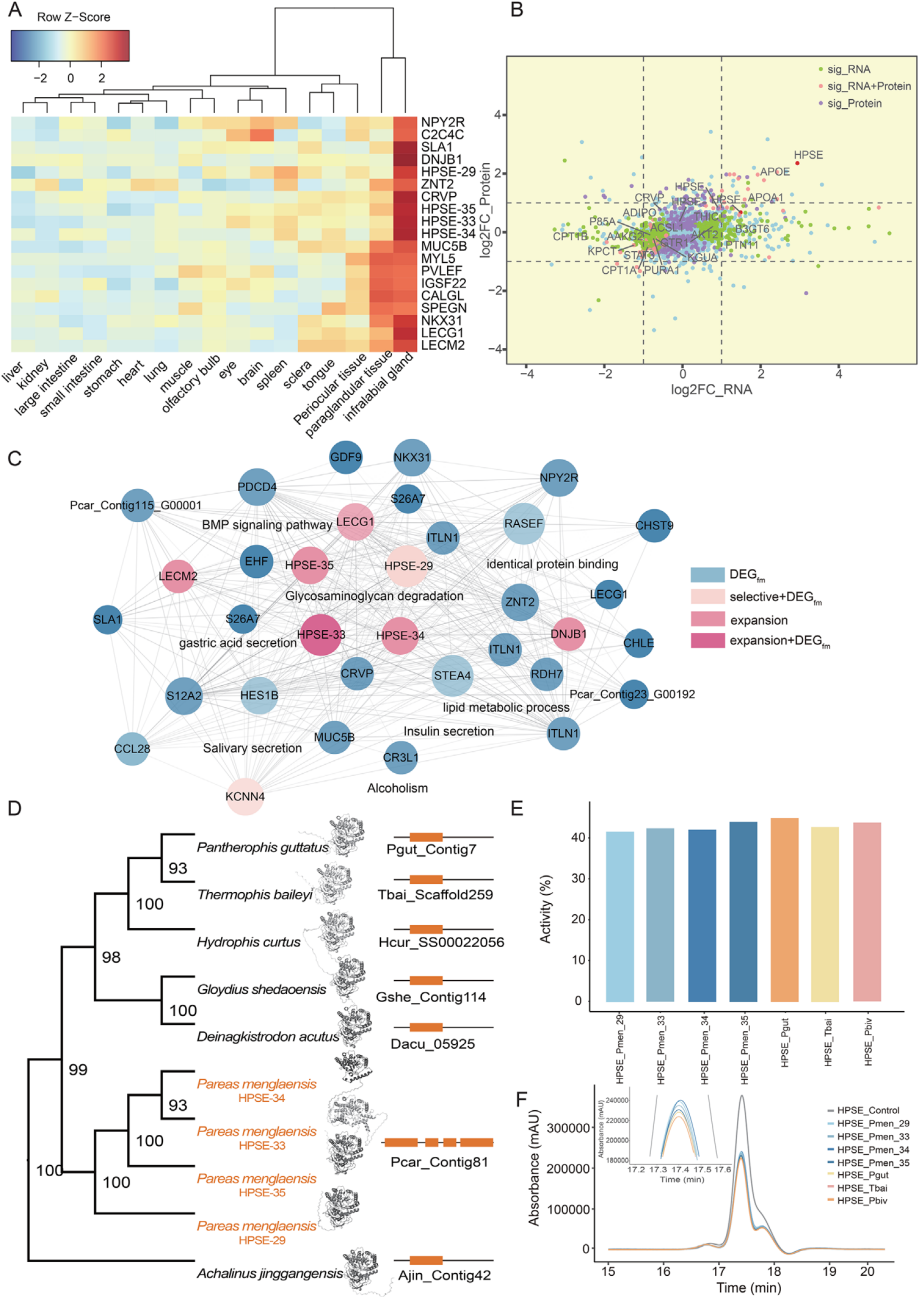
Infralabial gland‐specific gene expression associated with dietary specialization. A) Heatmap of differentially expressed genes in infralabial gland compared to 16 other tissues, highlighting 19 genes with highest up‐regulation. Each tissue sample included at least three biological replicates. Raw counts were normalized, averaged, and transformed into Z‐scores. B) Scatter plot illustrating expression changes in infralabial gland at mRNA and protein levels before and after snail feeding (*n* ≥ 3). Genes with significant (adjusted *p* < 0.05) differential expression at the transcriptional level (sig_RNA), protein level (sig_Protein), or both (sig_RNA+Protein), are marked in green, purple, and pink, respectively. C) Co‐expression network of hub genes (dark blue) strongly positively correlated with infralabial gland in *P. menglaensis*, categorized by additional characteristics: DEG_fm_ (light blue), differentially expressed hub genes in infralabial gland before and after feeding; selective + DEG_fm_ (pink), positively selected or rapidly evolving in *P. menglaensis*; expansion (light magenta), expanded in *P. menglaensis*; expansion + DEG_fm_ (magenta). D) Phylogenetic tree of *HPSE*s from *P. menglaensis* and other snakes, showing multiple copies of *HPSE* unique to *P. menglaensis*. Size of orange blocks on the right is proportional to the size of genes. E) Bar graph depicting comparable enzyme activities between four recombinant *P. menglaensis* HPSEs and three recombinant HPSEs from non‐snail‐eating snakes. Activity reflects the percentage of substrate hydrolyzed by each enzyme over a 4.5 h period. F) Chromatographic profiles of substrate degradation, illustrating differential reductions in peak areas among recombinant HPSEs. Colored traces correspond to active enzymes; gray traces denote heat‐inactivated controls.

To identify the role of the infralabial gland in the dietary adaptation of *P. menglaensis* to snail consumption, pairwise tissue comparisons were conducted. High‐quality RNA sequencing (RNA‐seq) data were obtained from the transcriptomes of the infralabial gland and 16 other tissues. A total of 2830 genes were identified as differentially expressed in the infralabial gland (**Figure** **3**A, Table S16, Supporting Information). These genes displayed significant enrichment in categories associated with glycosaminoglycan metabolism (e.g., hsa00531: glycosaminoglycan degradation, GO:0030207: chondroitin sulfate catabolic process and hsa00534: glycosaminoglycan biosynthesis – heparan sulfate/heparin), secretion of digestive juices (e.g., hsa04970: salivary secretion and hsa04971: gastric acid secretion), and lipid metabolism (e.g., hsa04975: fat digestion and absorption, hsa04979: cholesterol metabolism and hsa01212: fatty acid metabolism) (Table S17, Supporting Information). Among the differentially expressed genes, 15 originated from the 64 expanded gene families (Table S14, Supporting Information). Of these, nine were functionally annotated. Three corresponded to *HPSE*, which ranked among the most highly up‐regulated genes (Figure 3A). Two encoded C‐type lectin homologs (*LECG1* and *LECM2*), one of which (*LECG1*) is originally identified in many‐banded krait venom and known to induce blood cell aggregation.^[^ ^24^ ^]^ Two *DNJB1*s, which encode members of the DnaJ/Hsp40 family that function as molecular chaperones, promoting protein folding and enhancing fatty acid oxidation to alleviate insulin resistance.^[^ ^25^ ^]^ One *ZN135* encodes a zinc finger protein associated with regulation of cell morphology and cytoskeletal organization.^[^ ^26^ ^]^ The final annotated gene, *CEAM3*, is possibly involved in cell adhesion.^[^ ^27^ ^]^

Comparative transcriptomic and proteomic analyses of the infralabial gland before and after snail feeding identified 139 differentially expressed genes and 394 differentially expressed proteins (Tables S18 and S19, Supporting Information). Functional enrichment revealed significant overrepresentation of pathways related to glycosaminoglycan metabolism (hsa00532) and lipid metabolism (e.g., hsa04975 and hsa04979) (Tables S20 and S21, Supporting Information). Notably, *HPSE*, *APOE*, and *APOA1* exhibited further up‐regulation at the protein levels, or at both the transcript and protein levels, in the infralabial gland following feeding (Figure 3B, Tables S18 and S19, Supporting Information).

Weighted gene co‐expression network analysis (WGCNA) identified modules of co‐expressed genes associated with the specialized function of the infralabial gland in *P. menglaensis*, many of which exhibited species‐specific evolutionary signals (Figure 3C; Figure S4, Tables S22 and S23, Supporting Information). Beyond the previously discussed expanded *LECG1*, *LECM2*, *HPSE*, and *DNJB1* genes, additional genes showed signatures of positive selection or rapid evolution. Among them, *MUC16*, classified as both positively selected and rapidly evolving, encodes a membrane‐bound mucin implicated in the formation of protective lubricating barriers at mucosal surfaces to defend against particulate and pathogenic challenges (Table S14, Supporting Information).^[^ ^28^ ^]^

Given the species‐specific expansion, intact structural integrity and postprandial up‐regulation of *HPSE* variants in *P. menglaensis* (Figure 3D; Figures S5 and S6, Supporting Information), their enzymatic function was further assessed. Seven recombinant HPSE proteins, including four derived from *P. menglaensis* and three derived from non‐snail‐eating snakes (*Pantherophis guttatus*, *Thermophis baileyi*, and *Python bivittatus*), were expressed using a prokaryotic system and evaluated for their capacity to hydrolyze heparin sodium, a glycosaminoglycan substrate susceptible to cleavage by HPSE.^[^ ^29^, ^30^ ^]^ Gel permeation chromatography revealed that the *P. menglaensis* HPSE variants exhibited enzymatic activities comparable to those of HPSEs from non‐snail‐eating species (Figure 3E,F).

### 2.4 Gut Microbiomes Assist Snail Eating

**Figure 4:**
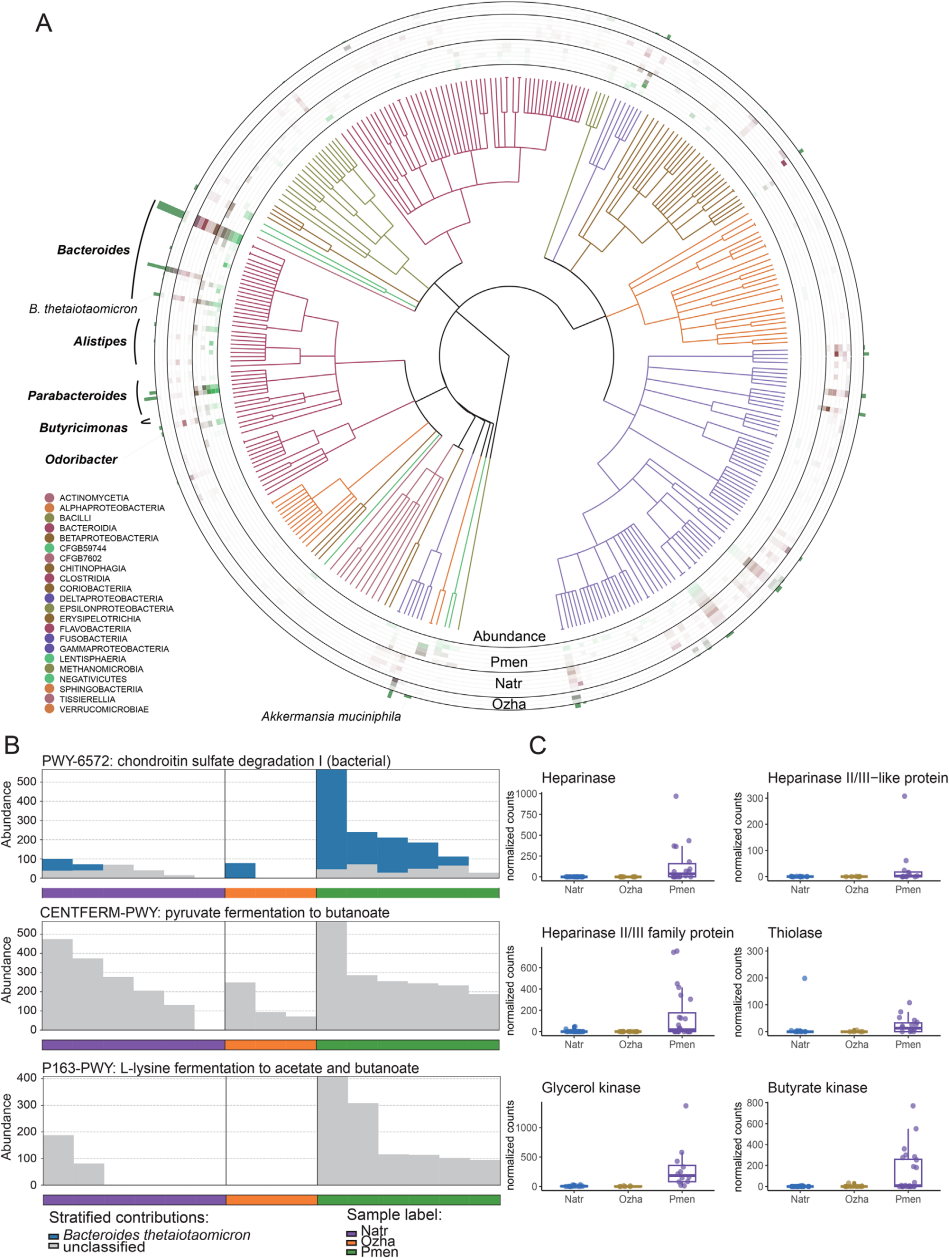
Adaptations of *Pareas menglaensis* gut microbiota for snail eating. A) Phylogeny of gut commensals detected in *P. menglaensis* and two non‐snail‐eating snakes. Bacteria from different classes are represented using different colors. From inner to outer rings, Circos plot shows the detected microbes, with specific microbe abundance in each sample represented by different colors (deeper color indicating higher abundance) and bars representing sum of microbial abundance in all samples. Dominant microbiota in *P. menglaensis* are denoted along the outer rim. B) Microbial pathways significantly (*p* < 0.05) enriched in gut symbionts with higher abundance in *P. menglaensis* (Pmen) compared to non‐snail‐eating snakes (Natr, Ozha). Bar charts show abundance of microbes in six *P. menglaensis* (green box), six *Naja atra* (purple box), and three *Opisthotropis zhaoermii* samples (orange box). C) Boxplots showing significant differences (adjusted *p* < 0.05) in abundance of six microbial genes associated with glycosaminoglycan or lipid metabolism among six *P. menglaensis*, six *N. atra*, and three *O. zhaoermii* individuals. *P*‐values were calculated using one‐sided Dunn's test with Benjamini‐Hochberg correction, following a significant Kruskal‐Wallis test. The whiskers indicate 1.5 times of the interquartile range.

As diet is recognized as the principal determinant of gut microbiome composition in snakes,^[^ ^31^ ^]^ the influence of dietary specialization in *P. menglaensis* was investigated through comparative metagenomic analysis. Gut metagenomes from six Mengla snail‐eating snakes were compared with those of two non‐snail‐eating species: the Chinese cobra (*Naja atra*) and Zhao's mountain stream snake (*Opisthotropis zhaoermii*). The gut microbiota of *P. menglaensis* exhibited greater intra‐species consistency and higher microbial diversity than those of the non‐snail‐eating snakes (Figure S7 and Table S24, Supporting Information). Among the 10 most abundant gut microbial taxa in *P. menglaensis*, *Bacteroides* was the most dominant genus, present in both comparator species (**Figure** **4**A; Figure S8, Supporting Information), although species‐level composition varied (Figure 4A). Notably, *B. thetaiotaomicron* and *B. fragilis* predominated in *P. menglaensis*, whereas *B. neonati* and *B. fragilis* dominated in the non‐snail‐eating snakes (Figure S8, Supporting Information)*. Parabacteroides* ranked second in abundance in *P. menglaensis*, while comprising only a minor fraction in *O. zhaoermii* (Figure 4A; Figure S8, Supporting Information). Additionally, *Alistipes*, *Butyricimonas*, and *Odoribacter*, though less abundant overall, were found to be dominant exclusively in *P. menglaensis* (Figure 4A; Figure S8, Supporting Information).

These taxonomic differences in the composition of dominant gut microbes likely contributed to the observed functional divergence in gut microbiome activity across species (Figure 4B). Glycosaminoglycan degradation (PWY‐6572: chondroitin sulfate degradation I) was significantly enriched in the microbiota of *P. menglaensis*, driven primarily by the high abundance of *B. thetaiotaomicron*, which was the second‐most abundant *Bacteroides* species in *P. menglaensis* but only marginally represented in the other two snakes (Figure 4B; Figure S8 and Table S25, Supporting Information). Consistently, genes encoding enzymes responsible for heparin and heparan sulfate degradation (e.g., heparinase and heparinase II/III family proteins) exhibited markedly higher levels in *P. menglaensis* (Figure 4C, Table S26, Supporting Information).^[^ ^32^, ^33^ ^]^

In addition, several microbial pathways associated with short‐chain fatty acid (SCFA) production were significantly enriched, including P163‐PWY: L‐lysine fermentation to acetate and butanoate, CENTFERM‐PWY: pyruvate fermentation to butanoate (Figure 4B; Table S25, Supporting Information). In addition, *P. menglaensis* exhibited robust expression of lipid‐metabolizing microbial enzymes (Figure 4C, Table S26, Supporting Information), notably butyrate kinase and thiolase, which are involved in key steps of SCFA butyrate production,^[^ ^34^ ^]^ and glycerol kinase, a critical enzyme involved in triacylglycerol synthesis and lipid storage.^[^ ^35^ ^]^ In contrast, microbial pathways associated with lipid breakdown, including FAO‐PWY: fatty acid and beta‐oxidation I (generic) and PWY‐5138: fatty acid and beta‐oxidation IV (unsaturated, even number), were significantly enriched in non‐snail‐eating species (Figure S9 and Table S25, Supporting Information). This pattern may reflect the higher abundance of lipophilic bacterial taxa such as *Aeromonas*, *Citrobacter*, and *Morganella* in those species (Figure S8, Supporting Information).^[^ ^36^, ^37^, ^38^ ^]^ Correspondingly, microbial genes involved in lipid catabolism were also significantly more abundant in these snakes (Figure S9 and Table S26, Supporting Information).

## 3 Discussion

Although snakes exhibit a wide diversity of dietary habits, the genetic and physiological mechanisms underlying these adaptations remain largely underexplored. Their varied feeding habits make snakes an ideal model for studying dietary specialization. This study provides insight into the molecular and symbiotic adaptations supporting snail‐eating in an oligophagous snake species, focusing on genetic modifications in glycosaminoglycan and lipid metabolism and the complex interactions between host and gut symbionts that facilitate digestion and nutrient utilization.

Pareid snakes face considerable challenges when preying on snails, including extracting the body from the shell and digesting the substantial quantities of mucus secreted by the snail. To overcome these mechanical and physiological challenges, *P. menglaensis* has evolved specialized infralabial glands exhibiting high expression of multiple toxin homologs. These include *SLA1*, homologous to the agglutinin of the hundred‐pace snake; *LECG1*, homologous to a *Bungarus*‐derived C‐type lectin; and *CRVP*, homologous to the cysteine‐rich venom protein found in Lichtenstein's green racer. In venom systems, the agglutinin and C‐type lectin are known to promote erythrocyte aggregation,^[^ ^24^, ^39^ ^]^ while the cysteine‐rich venom proteins can hinder muscle contraction in prey.^[^ ^40^ ^]^ The expression of these toxin homologs in the infralabial gland may facilitate prey immobilization and prevent retraction into the shell, thereby enabling more efficient access to the snail body. However, direct functional validation of these putative roles is required in future studies.

Beyond these toxin homologs, additional genes expressed in the infralabial gland appear to have undergone adaptive changes to facilitate snail eating. For example, *DNJB1*, a DnaJ/Hsp40 family member, was expanded and may contribute to correct folding of glycosaminoglycan‐degrading enzymes such as HPSE. *MUC16*, which encodes a large mucin involved in mucosal barrier formation, showed evidence of both positive selection and accelerated evolution, suggesting an adaptive role in protecting oral tissues from abrasion during snail ingestion.

These genomic innovations were accompanied by functionally relevant gene family contractions, which may reflect trade‐offs associated with dietary specialization. Contraction of TRIM family genes—key regulators of lipid metabolism and insulin sensitivity—has been linked to metabolic dysregulation, including hepatic lipid accumulation and insulin resistance,^[^ ^17^, ^18^ ^]^ potentially constraining the ability of *P. menglaensis* to efficiently process high‐fat prey such as rodents. Similarly, contractions in olfactory receptor genes may impair lipid‐responsive pathways, including fatty acid oxidation and hormone‐sensitive lipase activation,^[^ ^19^, ^20^ ^]^ further reducing metabolic flexibility. In parallel, contraction of immune‐related genes may compromise the snake's capacity to manage diverse pathogens encountered in generalist diets. These converging constraints, including metabolic inefficiency, impaired lipid processing, and immune vulnerability, likely reinforce the narrow trophic niche of *P. menglaensis*, despite the availability of alternative prey in its environment.

**Figure 5:**
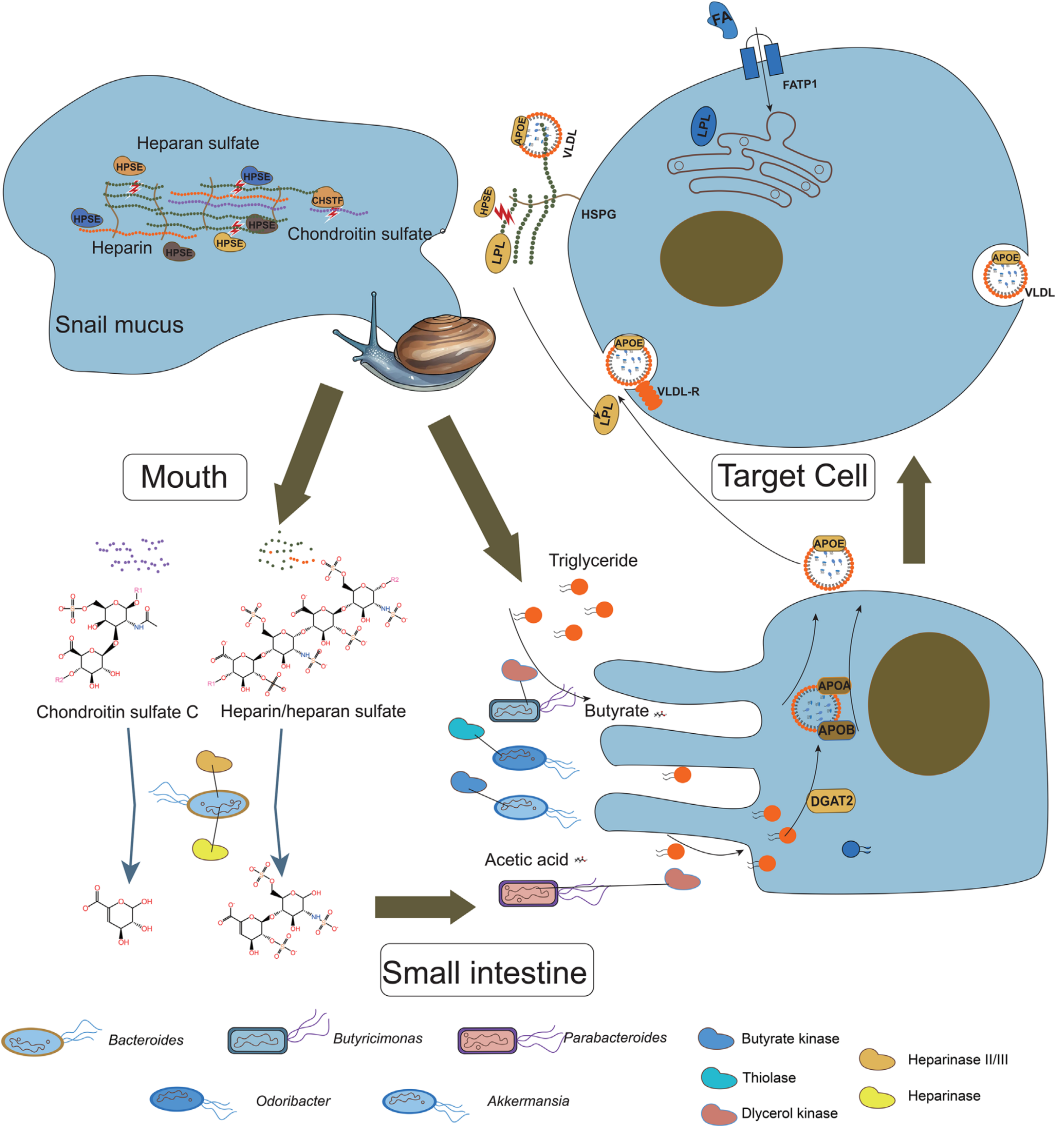
Hologenomic adaptation underlying dietary specialization in *Pareas menglaensis*. Adaptational changes in the snake genome and gut microbiota have provided solutions to challenges such as lipid shortages and mucus degradation encountered during snail consumption.

Snail mucus, rich in glycosaminoglycans, presents a major biochemical obstacle to digestion due to its adhesive and protective properties (**Figure** **5**).^[^ ^41^, ^42^ ^]^ Our results revealed a coordinated host‐microbiome interaction facilitating glycosaminoglycan breakdown. Genes associated with glycosaminoglycan metabolism were highly expressed in the infralabial gland of *P. menglaensis*, with many showing signs of rapid evolution (Figure 2 and Figure 3). In particular, *HPSE* underwent species‐specific expansion in *P. menglaensis*, with four functional paralogs expressed in the infralabial gland. Although enzymatic activity showed no significant differences compared to other snakes, this genomic expansion likely enhanced total enzymatic output through a gene dosage effect, enabling more efficient degradation of glycosaminoglycans. The postprandial up‐regulation of HPSE provided further evidence of its pivotal role in enabling efficient mucus degradation and facilitating dietary adaptation in *P. menglaensis* (Figure 5).

Additionally, *P. menglaensis* displayed a notably higher abundance of glycosaminoglycan‐degrading bacteria and increased abundance of glycosaminoglycan‐degrading enzymes in its gut microbiome compared to snakes with different dietary patterns (Figure 4 and Figure 5). Previous studies have shown that vampire bats primarily rely on *Clostridium*, *Streptococcus*, and *Staphylococcus* to degrade heparan sulfate, aiding in blood digestion. In *P. menglaensis*, however, neither *Streptococcus* nor *Staphylococcus* were present, and *Clostridium* showed low abundance. Interestingly, both vampire bats and Mengla snail‐eating snakes appear to employ heparinase II/III‐like or heparinase II/III family proteins produced by their gut symbionts for heparan sulfate degradation.^[^ ^11^ ^]^ This represents an intriguing example of convergent evolution across mammalian and reptilian lineages, where similar microbial functions have evolved to support comparable dietary requirements.

Our findings also highlight a coordinated interaction between the host genome and microbiome in nutrient utilization, enabling the Mengla snail‐eating snake to cope with the limited lipid content characteristic of a snail‐based diet. Snails are inherently low in lipid content,^[^ ^9^ ^]^ which likely drives the selection of genes involved in lipid metabolism within *P. menglaensis*. For example, *APOE*, a gene involved in the regulation of plasma and tissue lipid levels,^[^ ^43^, ^44^, ^45^ ^]^ was found to be under positive selection and harbored *P. menglaensis*‐specific mutations. These mutations conferred a distinct cholesterol‐binding profile, likely enhancing lipid‐binding capacity. Although mutations in the LDLR‐binding region of *P. menglaensis* APOE did not significantly alter binding affinity—possibly due to conformational flexibility introduced by extensive random coils in LDLRs during APOE‐LDLR interaction simulations—the optimal binding model suggested increased binding stability associated with the mutated APOE variant (Figure S3, Supporting Information). APOE also binds to cell surface glycosaminoglycans, such as heparin, which cooperate with LDLRs to mediate APOE‐bound lipid uptake. No mutations were observed at sites previously shown to be critical for APOE‐glycosaminoglycan interactions (Figure S10, Supporting Information).^[^ ^46^ ^]^ Overall, these results suggest that APOE mutations in *P. menglaensis* likely enhance lipid binding and improve lipid delivery to metabolically demanding tissues, while preserving the efficiency of glycosaminoglycan‐mediated uptake (Figure 5).

Possibly reflecting the low‐fat diet, gut microbes that favor high‐fat environments were significantly less abundant in *P. menglaensis*, as were microbial genes associated with lipid degradation (Figures S8 and S9, Supporting Information). However, taxa with beneficial metabolic functions were significantly enriched in response to the dietary limitations of snail consumption (Figure 4 and Figure 5). The low‐fat dietary context may promote the proliferation of *Akkermansia muciniphila*,^[^ ^47^ ^]^ a bacterium that degrades mucins to produce SCFAs that can be used as an energy source by the host.^[^ ^48^ ^]^ Its greater abundance in *P. menglaensis* relative to other snakes may confer metabolic advantages (Figure 4; Figure S8, Supporting Information). *Butyricimonas*, *Odoribacter*, and *Parabacteroides*, key producers of SCFAs, were observed at markedly higher abundances in *P. menglaensis* compared to other snakes (Figure 4; Figure S8, Supporting Information). *Butyricimonas* and *Odoribacter* primarily produce butyrate, a key regulator of energy homeostasis,^[^ ^49^, ^50^, ^51^ ^]^ while *Parabacteroides* predominantly secretes acetate, which supports host energy metabolism and alleviates inflammatory responses.^[^ ^52^ ^]^ The *P. menglaensis* gut microbiota likely utilize oligosaccharides derived from glycosaminoglycan degradation to sustain SCFA production as an energy source (Figure 5).^[^ ^53^, ^54^ ^]^ Furthermore, the significantly higher expression of microbial genes, such as butyrate kinase and thiolase, further suggests active SCFA production, particularly butyrate, within the intestines of *P. menglaensis* (Figure 4C; Table S26, Supporting Information). High expression of glycerol kinase may help compensate for the lipid deficiency inherent in a snail‐based diet by enhancing fatty acid utilization (Figure 5). Interestingly, enzymes involved in butyrate production and glycerol kinase are also enriched in the gut microbiota of the common vampire bat, thought to be important in energy storage.^[^ ^11^ ^]^ This parallel adaptation suggests convergent evolutionary strategies for managing nutrient scarcity between vampire bats and Mengla snail‐eating snakes, both of which rely on lipid‐poor diets.

Mengla snail‐eating snakes may also need to mitigate the accumulation of ammonia, a toxic byproduct of digesting protein‐rich snails.^[^ ^55^ ^]^ While uric acid typically serves as the primary nitrogenous waste in most reptiles, some species may increase urea excretion under higher protein intake.^[^ ^56^ ^]^ In this study, genes such as *SLC14A2* and *ASS1* exhibited rapid evolution in *P. menglaensis*, suggesting adaptations to its protein‐rich diet. These genes contribute to the urea cycle, which detoxifies ammonia by converting it to urea, thereby minimizing ammonia toxicity.^[^ ^57^, ^58^, ^59^ ^]^ Complementing this, *Alistipes*, a genus known for its ureolytic capacity,^[^ ^60^ ^]^ emerged as one of the most abundant bacterial symbionts in *P. menglaensis* (Figure 4; Figure S8, Supporting Information). However, unlike the common vampire bat, which relies on microbial urease for urea degradation, *P. menglaensis* did not exhibit elevated expression of microbial urease genes, possibly because urea is not its primary nitrogenous waste.^[^ ^11^ ^]^

## 4 Conclusion

Studies of dietary adaptations in animals through a holobiome perspective remain limited outside of mammals. This research highlights the integrative relationship between the genome and microbiome in driving dietary adaptations in the Mengla snail‐eating snake and presents evidence of functional convergence between mammals and reptiles in gut symbionts. Overall, these findings extend our understanding of dietary adaptation mechanisms across species and underscore the pivotal role of the hologenome in evolutionary processes.

## 5 Experimental Section

### Ethics Approval

All experimental protocols were approved by the Animal Research and Ethics Committee of the Chengdu Institute of Biology, Chinese Academy of Sciences (CIBDWLL2023011).

### Histomorphology of the *P. Menglaensis* Infralabial Gland

Histomorphological analysis was conducted following the methods of Wang et al. (2022).^[^ ^10^ ^]^ In brief, the snakes were euthanized via intraperitoneal injection of sodium thiopental (Table S27, Supporting Information). The entire head was severed at the first cervical vertebra and skinned, after which the infralabial glands were individually dissected for histological and histochemical analysis. The head section and one of the infralabial gland sections were stained using hematoxylin‐eosin staining for general observations. To differentiate secretory cell types, the remaining infralabial gland sections were subjected to Coomassie brilliant blue R250 staining, Alcian blue (AB) pH 2.5 staining, periodic acid‐Schiff (PAS) staining, or a combined PAS and AB reaction. All photomicrographs were obtained using a Cnoptec B302 microscope connected to a digital camera (Sony ICX285AQ CCD) and image acquisition software (ImageView). For ultrastructural observations, fresh tissue was cut into 1‐mm^3^ blocks, then fixed and embedded in resin (EMBed 812). Ultrathin sections (60–80 nm) were prepared using an ultramicrotome and observed under a transmission electron microscope.

### Gene Family Analysis and Functional Enrichment

OrthoFinder v2.5.4 was used to identify multi‐copy and single‐copy gene families in *P. menglaensis* and other snake species with different feeding habits.^[^ ^61^, ^62^ ^]^ A maximum‐likelihood tree based on all single‐copy genes was reconstructed using IQTree v1.6.520^[^ ^63^ ^]^ with parameters: ‐nt 10 ‐st DNA ‐bb 1000 ‐alrt 1000. Based on the phylogeny, PAML v4.9i^[^ ^64^ ^]^ was utilized to detect positively selected genes (PSGs) and genes with high molecular evolutionary rates (REGs). RELAX^[^ ^65^ ^]^ and FEL model in HyPhy v2.5.48^[^ ^66^ ^]^ were used to validate the PSGs and REGs. CAFE v4.2.1^[^ ^67^ ^]^ was used to detect significantly expanded or contracted gene families.

The KOBAS v3.0.3^[^ ^68^ ^]^ database was used to assign Gene Ontology (GO) terms, Kyoto Encyclopedia of Genes and Genomes (KEGG) Orthology (KO) identifiers and Reactome pathway identifiers to each gene.^[^ ^69^, ^70^, ^71^ ^]^ GO and Reactome pathway enrichment analyses were conducted using the R package clusterProfiler v4.0.1,^[^ ^72^ ^]^ with GO and Reactome pathway terms showing a *p* < 0.05 considered significantly enriched. For KEGG enrichment analysis, KO identifiers were first mapped using the KEGG Mapper Reconstruct tool (https://www.genome.jp/kegg) as described in Yan et al. (2022).^[^ ^73^ ^]^ Counts of KO identifiers for foreground and background genes within each reconstructed pathway were then analyzed using a hypergeometric test, with KEGG pathways showing a *p* < 0.05 deemed significantly enriched.

### Transcriptome Sequencing and Assembly

Six *P. menglaensis* individuals were euthanized via rapid decapitation and subsequently dissected (Table S27, Supporting Information). Various tissues were collected, including infralabial glands, paraglandular tissue, heart, muscle, liver, lung, kidney, brain, eyes, stomach, small intestine, large intestine, spleen, olfactory bulb, tongue, sclera, and periocular tissue, then flash‐frozen in liquid nitrogen and stored in a refrigerator at −80 °C. Total RNA was extracted from each sample using a QIAGEN RNA Mini Kit, followed by RNA quality assessment. cDNA libraries were constructed and sequenced on the Illumina NovaSeq 6000 platform in PE150 mode. Quality‐controlled RNA‐seq reads were aligned to the *P. menglaensis* genome using STAR v2.7.6a^[^ ^74^ ^]^ with the parameters: chimSegmentMin 2; outFilterMismatchNmax 3. Gene and transcript expression levels were quantified using RSEM v1.2.28.^[^ ^75^ ^]^

Seven additional snakes were assigned to two groups, each comprising at least three individuals (Table S28, Supporting Information). In one group, infralabial glands were sampled without prior feeding, while in the second group, infralabial glands were sampled within 30 min of feeding on snails. Differentially expressed genes (DEGs) between the two groups were identified using the methods described below, followed by functional enrichment analysis.

### Analysis of DEGs

DESeq2 v1.34.0^[^ ^76^ ^]^ and edgeR v3.36.0^[^ ^77^ ^]^ were used for differential expression analysis to identify DEGs across tissue types. Genes with an adjusted *p* < 0.05 and a log2 fold change > 1 were classified as differentially expressed. Enrichment analysis of DEGs was performed using KOBAS v3.0.3 and clusterProfiler v4.0.1.

### Correlation Analysis of Tissue Transcriptomes

Co‐expressed gene networks significantly correlated with specific tissue types were identified using the WGCNA package in R.^[^ ^78^ ^]^ Unsigned co‐expression networks were constructed using a selected soft threshold (power = 5). To identify modules of highly connected RNAs, similar networks were merged using the parameters: minModuleSize = 30, pamRespectsDendro = FALSE, mergeCutHeight = 0.25. Modules with a correlation coefficient > 0.90 and *p* < 0.05 for each tissue were selected for further analysis. Functional enrichment analysis of genes within these modules was performed as previously described. Hub genes within the co‐expression network were identified using Cytoscape v3.7.2^[^ ^79^ ^]^ based on degree centrality.

### Comparative Proteomic Analysis of Infralabial Gland Before and After Snail Feeding

Proteomic profiling was conducted on the infralabial glands of the seven previously described snakes (Table S28, Supporting Information). Following sample isolation at 4 °C, tissues were dissected into small pieces after removal of blood, fat, and connective tissue. The processed samples were immediately frozen in liquid nitrogen and stored at −80 °C until further use. The frozen tissues were ground in liquid nitrogen and lysed using a lysis buffer with an ultrasonic processor. After centrifugation to remove cell debris, protein concentration was determined using a BCA Assay Kit.

Equal amounts of protein from each sample were reduced and alkylated with 5 mM dithiothreitol and 11 mM iodoacetamide, respectively, then subjected to trypsin digestion. An initial digestion was performed overnight at a trypsin‐to‐protein mass ratio of 1:50, followed by a second digestion for 4 h at a 1:100 mass ratio. The peptides were subsequently desalted using a C18 SPE column.

The digested samples were subjected to liquid chromatography‐tandem mass spectrometry (LC‐MS/MS). First, tryptic peptides were dissolved in solvent A (0.1% formic acid, 2% acetonitrile/in water) and loaded onto a home‐made reversed‐phase analytical column (25 cm length, 75/100 µm i.d.). Peptide separation was performed using the EASY‐nLC 1200 Ultra‐High Performance Liquid Chromatography system, with the following gradient: 0–14.5 min, 6%–22% solvent B; 14.5–17.5 min, 22%–34% solvent B: 17.5–19 min, 34%–80% solvent B: 19–20 min, 80% solvent B, at a constant flow rate of 700 nL min^−1^. The separated peptides were then analyzed using an Orbitrap Exploris 480 with a nano‐electrospray ion source, applying an electrospray voltage of 2 300 V. The FAIMS compensation voltage was set to −45 V. Precursors and fragments were detected at the Orbitrap, with a full MS scan resolution of 60 000 over a 350–1 400 m/z range. The MS/MS scans were acquired with a fixed first mass of 120.0 m z^−1^ at a resolution of 15 000. Higher‐energy collisional dissociation fragmentation was conducted using a normalized collision energy of 27%, with an automatic gain control target of 1 × 10^6^ and maximum injection time of 22 ms.

Subsequent analyses were conducted based on the raw MS data. The DIA‐NN search engine v1.8^[^ ^80^ ^]^ was utilized to match tandem mass spectra against all protein‐coding sequences annotated in the *P. menglaensis* genome, concatenated with the reverse decoy database. Trypsin/P was specified as the cleavage enzyme, allowing up to one missed cleavage, with N‐terminal Met excision and carbamidomethylation on Cys as fixed modifications, and a false discovery rate set to < 1%. Relative protein quantities from each group were averaged, and differentially expressed proteins between groups were identified using a *t*‐test, with the significance threshold set at *p* < 0.05. Functional enrichment analysis of differentially expressed proteins was performed using the methods previously described.

### Identification of Species‐Specific Mutations of APOE

Previously identified single‐copy genes were analyzed to detect *P. menglaensis‐*specific mutations using custom Python scripts, and the functional impacts of these mutations were predicted using PROVEAN v1.1.5.^[^ ^81^ ^]^ Mutations with a PROVEAN score < −2.5 were predicted to affect protein function. The 3D structure of APOE was modeled using AlphaFold v2.0.0 with the CASP14 model.^[^ ^82^ ^]^ Pymol v2.6^[^ ^83^ ^]^ was employed to examine structural differences between the mutated and reference versions of the protein.

To evaluate ligand binding, structural files of the mutated *P. menglaensis* APOE, its non‐mutated counterpart from non‐snail‐eating snakes, and a potential ligand were loaded into Dockey v1.0.1.^[^ ^84^ ^]^ Dockey simulated the binding interaction 10 times, generating parameters for model fitness, binding stability and affinity. These metrics were then compared between the APOE binding models from *P. menglaensis* and those from snakes with alternative diets. To overcome Dockey's limitations in handling high‐complexity protein‐protein interactions, we employed AlphaFold v3.0.0^[^ ^85^ ^]^ to resolve distinct binding architectures between APOE‐LDLR interactions. It was also used to validate the APOE‐cholesterol binding architectures.

### Metagenomic Sequencing and Assembly

Six Mengla snail‐eating snakes and three Zhao's mountain stream snakes were captured in the wild during visual encounter surveys. When a snake was opportunistically located, it was captured using tongs and safely secured in a clear plastic box. The Mengla snail‐eating snakes were sampled from Xishuangbanna, Yunnan Province, within three days, and the Zhao's mountain stream snakes were sampled from Leishan, Guizhou Province, within three days (Table S29, Supporting Information). Six Chinese cobras were collected together at one time from Sichuan Province; however, since they were donated by villagers, the exact capture locations were unknown. All individuals of the three snake species were collected during summer.

These snakes were sacrificed by decapitation, and their fresh gut contents were immediately collected and stored at −80 °C with the same procedures. DNA was extracted from the gut microbiome using a TIANamp Stool DNA Kit (TIANGEN) following the manufacturer's instructions. DNA quality was verified prior to constructing sequencing libraries using a DNA Library Prep Kit (Yeasen). Libraries were sequenced on the DNBSEQ‐T7 system in PE150 mode, generating a median of 7.79 Gb of sequencing data.

### Metagenomic Taxonomic Annotation and Functional Analysis

The resulting metagenomic data were imported into metaWRAP^[^ ^86^ ^]^ for further analysis. The MetaWRAP read_qc module was used to trim the reads and remove host contamination. Quality‐checked reads were then co‐assembled using the metaWRAP Assembly module. The Kraken module was applied to annotate the co‐assembly results, enabling identification of community composition, while microbial abundance across taxonomic levels was estimated using Bracken v2.9.^[^ ^87^ ^]^ Alpha and beta diversity were then estimated using the EasyMetagenome pipeline.^[^ ^88^ ^]^

The co‐assembly was then binned with the Binning module, using the CONCOCT, MaxBin, and metaBAT algorithms. The generated bins were consolidated into a single bin set (‐c 70 ‐x 5) via the Bin_refinement module. The Reassemble_bins module was then used to further refine the consolidated bin set, with taxonomic and functional annotations determined for each bin using the Classify_bins and Annotate_bins modules, respectively. Functional enrichment of gut microbes significantly more abundant in *P. menglaensis* compared to other snakes was performed with MetaPhlAn^[^ ^89^ ^]^ following the biobakery_workflows. Microbial gene annotation of identified bins was conducted with DRAM,^[^ ^90^ ^]^ and reads were mapped to gene transcripts using STAR, with microbial gene abundance levels estimated via RSEM. To compare gene expression between *P. menglaensis* and non‐snail‐eating snakes, gut microbial genes from all species were first clustered using OrthoFinder, and read counts of each cluster were calculated. A Kruskal‐Wallis test was then used to assess significance of differences in microbial gene abundance among *P. menglaensis*, *N. atra and O. zhaoermii*. Dunn's test was employed to conduct pairwise comparisons following a statistically significant difference detected by the Kruskal‐Wallis test. Genes exhibiting significantly different abundance between *P. menglaensis* and non‐snail‐eating snakes were defined with a Benjamini‐Hochberg adjusted *p* < 0.05.

### Acquisition of Recombinant P. Menglaensis HPSEs

Recombinant protein expression and purification were performed by Gene Universal Inc. In brief, the *HPSE* gene was amplified via polymerase chain reaction (PCR) using primers with overhangs, followed by sequence verification. The amplified product was digested with NdeI and XhoI (SibStar) and cloned into the pET‐28a(+) vector (Gene Universal Inc.) (Table S30, Supporting Information). Heat shock transformation was then performed on competent *Escherichia coli* TOP10 cells (Gene Universal Inc.). Positive clones were screened via PCR, and plasmid DNA was extracted and further confirmed by sequencing. The extracted plasmid was also digested with ApaI and XhoI (SibStar) to ensure that the resulting bands matched the expected fragment sizes.

The extracted plasmid was then transformed into *E. coli* BL21(DE3) cells (Gene Universal Inc.), and positive clones were inoculated in Luria‐Bertani (LB) medium. Recombinant protein expression was induced with 0.5 mM IPTG at 37 °C for 4 h, with protein expression validated by sodium dodecyl‐sulfate polyacrylamide gel electrophoresis (SDS‐PAGE) and Western Blotting (Figure S11, Supporting Information). After assessing solubility, large‐scale production of recombinant proteins was conducted, followed by purification using Ni‐NTA affinity chromatography, with a PBS‐Urea (pH 7.4) buffer containing 500 mM imidazole. Purified proteins were confirmed by SDS‐PAGE (Figure S11, Supporting Information), refolded, and dialyzed in a PBS buffer containing 300 mM NaCl and 10% Glycerol at pH 7.4. Finally, the filtered recombinant proteins were stored at −80 °C until further use.

### Enzyme Activity Assays

Heparinolytic activity was quantified using a Shimadzu gel permeation chromatography (GPC) system equipped with a Shodex OHpak SB‐806M HQ column (8.0×300 mm). The mobile phase consisted of 0.1 M ammonium acetate buffer (pH 6.8) delivered at 0.6 mL min^−1^. Samples were filtered through 0.45 µm membranes prior to 25 µL injections. Recombinant HPSE proteins (0.4 mg mL^−1^ in PBS) from *P. menglaensis* and three non‐snail‐eating snakes were incubated with substrate solution containing 0.5 mg mL^−1^ heparin sodium (MACKLIN) in 10 mM PBS (pH 7.0) at a 100:1 (v/v) substrate‐to‐enzyme ratio. Reactions proceeded for 4.5 h at 37 °C followed by thermal inactivation at 100 °C for 10 min. Parallel control reactions used heat‐inactivated enzymes (100 °C, 10 min pretreatment). Chromatographic profiles were analyzed by monitoring absorbance at 210 nm. Enzyme activity was calculated as

(1) $$\mathrm{Activity}\;\left(\%\right)=\left(1-\frac{A_{\mathrm{sample}}}{A_{\mathrm{control}}}\right)\times 100$$

where A_sample_ and A_control_ represent the integrated peak areas of heparin degradation products in experimental and heat‐inactivated control groups, respectively.

### Statistical Analysis

For statistical analyses of results generated by Dockey and AlphaFold3, data were presented directly using boxplots. Dockey and AlphaFold3 simulated binding interactions 10 and 50 times, respectively. A two‐sided Student's *t*‐test was used to assess significant differences, with significance defined as *p* < 0.05. Statistical analyses were performed using R. For identifying differentially expressed proteins before and after feeding snails, A two‐sided Student's *t*‐test was conducted in R to determine significant differences, with an adjusted *p* < 0.05 considered significant. Each group included at least three individuals. For gut microbiome analyses, six *P. menglaensis*, six *N. atra*, and three *O. zhaoermii* individuals were analyzed. When comparing microbial gene abundance between *P. menglaensis* and non‐snail‐eating snakes, data were presented as boxplots. A Kruskal‐Wallis test was used to assess differences in microbial gene abundance among *P. menglaensis*, *N. atra*, and *O. zhaoermii*. Following a significant Kruskal‐Wallis result, one‐sided Dunn's tests were used for pairwise comparisons. Genes with significantly different abundances between *P. menglaensis* and the other two snakes were identified using Benjamini‐Hochberg‐adjusted *p* < 0.05. Statistical analyses were performed using R.

## Conflict of Interest

The authors declare the following competing interests that one patent has been registered (CN202510860383.8).

## Supporting information



Supporting Information

Supporting Information

## Data Availability

All assemblies, raw sequencing data, and analyzed data generated in this study are available in the National Genomics Data Center [https://ngdc.cncb.ac.cn/] under the BioProject number PRJCA032174.

## References

[1] S.‐C. Cheng , C.‐B. Liu , X.‐Q. Yao , J.‐Y. Hu , T.‐T. Yin , B. K. Lim , W. Chen , G.‐D. Wang , C.‐L. Zhang , D. M. Irwin , Z.‐G. Zhang , Y.‐P. Zhang , L. Yu , Natl. Sci. Rev. 2022, 10, nwac174. 37124465 10.1093/nsr/nwac174 PMC10139702

[2] Y. Hu , Q. Wu , S. Ma , T. Ma , L. Shan , X. Wang , Y. Nie , Z. Ning , L. Yan , Y. Xiu , F. Wei , Proc. Natl. Acad. Sci. USA 2017, 114, 1081. 28096377 10.1073/pnas.1613870114 PMC5293045

[3] M. Blumer , T. Brown , M. B. Freitas , J. A. Oliveira , A. E. Morales , T. Schell , C. Greve , M. Pippel , D. Jebb , N. Hecker , A.‐W. Ahmed , B. M. Kirilenko , M. Foote , A. Janke , B. K. Lim , M. Hiller , Sci. Adv. 2022, 8, abm6494. 10.1126/sciadv.abm6494 PMC8956264 35333583

[4] D. G. Broadley , Herpetologica 1979, 35, abm6494.

[5] K. Coleman , L. A. Rothfuss , H. Ota , K. V. Kardong , J Herpetol 1993, 27, 320.

[6] B. C. Jayne , H. K. Voris , P. K. Ng , Biol J Linn Soc 2018, 123, 636.

[7] J. K. Webb , R. Shine , W. R. Branch , P. S. Harlow , J. Zool. 2000, 250, 321.

[8] A. O'Hanlon , C. D. Williams , M. J. Gormally , J. Zool. 2019, 307, 203.

[9] S. Ghosh , C. Jung , V. B. Meyer‐Rochow , Ann Aquac Res 2016, 3, 1024.

[10] D. Wang , J. Ren , K. Jiang , Asian Herpetol. Res. 2022, 13, 1024.

[11] M. L. Zepeda Mendoza , Z. Xiong , M. Escalera‐Zamudio , A. K. Runge , J. Thézé , D. Streicker , H. K. Frank , E. Loza‐Rubio , S. Liu , O. A. Ryder , J. A. Samaniego Castruita , A. Katzourakis , G. Pacheco , B. Taboada , U. Löber , O. G. Pybus , Y. Li , E. Rojas‐Anaya , K. Bohmann , A. Carmona Baez , C. F. Arias , S. Liu , A. D. Greenwood , M. F. Bertelsen , N. E. White , M. Bunce , G. Zhang , T. Sicheritz‐Pontén , M. P. T. Gilbert , Nat. Ecol. Evol. 2018, 2, 659. 29459707 10.1038/s41559-018-0476-8 PMC5868727

[12] J.‐E. Ma , L.‐M. Li , H.‐Y. Jiang , X.‐J. Zhang , J. Li , G.‐Y. Li , J.‐P. Chen , FEBS Open Bio. 2018, 8, 1247. 10.1002/2211-5463.12461 PMC6070644 30087830

[13] K. V. Kardong , D. L. Luchtel , J. Morphol. 1986, 188, 1. 29966406 10.1002/jmor.1051880102

[14] S. Yoshie , M. Ishiyama , T. Ogawa , Arch. Histol. Jpn. 1982, 45, 375. 7165495 10.1679/aohc.45.375

[15] C. Peng , D.‐D. Wu , J.‐L. Ren , Z.‐L. Peng , Z. Ma , W. Wu , Y. Lv , Z. Wang , C. Deng , K. Jiang , C. L. Parkinson , Y. Qi , Z.‐Y. Zhang , J.‐T. Li , Cell 2023, 186, 2959. 37339633 10.1016/j.cell.2023.05.030

[16] Z. Wang , C. Peng , W. Wu , C. Yan , Y. Lv , J.‐T. Li , Sci. China Life Sci. 2023, 66, 2399. 37256419 10.1007/s11427-023-2362-5

[17] J. Zhang , Y. Zhang , Z. Ren , D. Yan , G. Li , Front Endocrinol (Lausanne) 2023, 14, 1210330. 37867509 10.3389/fendo.2023.1210330 PMC10585262

[18] J. Chen , X. Feng , X. Zhou , Y. Li , Diabetes Obes. Metab. 2024, 26, 3. 10.1111/dom.15294 37726973

[19] S. Zhang , L. Li , H. Li , Br. J. Pharmacol. 2021, 178, 4792. 34411276 10.1111/bph.15666

[20] C. Wu , T. T. Thach , Y. J. Kim , S. J. Lee , Biochim. Biophys. Acta Mol. Cell Biol. Lipids 2019, 1864, 489. 30639733 10.1016/j.bbalip.2019.01.004

[21] F. Gong , P. Jemth , M. L. E. Galvis , I. Vlodavsky , A. Horner , U. Lindahl , J.‐P. Li , J. Biol. Chem. 2003, 278, 35152. 12837765 10.1074/jbc.M300925200

[22] M. Fuller , P. J. Meikle , J. J. Hopwood , Glycobiology 2004, 14, 443. 14718373 10.1093/glycob/cwh049

[23] T. Kaneiwa , S. Mizumoto , K. Sugahara , S. Yamada , Glycobiology 2010, 20, 300. 19889881 10.1093/glycob/cwp174

[24] L. P. Lin , Q. Lin , Y. Q. C. Wang , Toxicon 2007, 50, 411. 17561224 10.1016/j.toxicon.2007.04.019

[25] A. Diane , H. Abunada , N. Khattab , A. S. M. Moin , A. E. Butler , M. Dehbi , Ageing Res. Rev. 2021, 67, 101313. 33676026 10.1016/j.arr.2021.101313

[26] S. W. Bai , M. T. Herrera‐Abreu , J. L. Rohn , V. Racine , V. Tajadura , N. Suryavanshi , S. Bechtel , S. Wiemann , B. Baum , A. J. Ridley , BMC Biol. 2011, 9, 54. 21834987 10.1186/1741-7007-9-54 PMC3201212

[27] S. Rebstock , K. Lucas , J. A. Thompson , W. Zimmermann , J. Biol. Chem. 1990, 265, 7872. 2335509

[28] X. Chen , I. K. Sandrine , M. Yang , J. Tu , X. Yuan , Front. Immunol. 2024, 15, 1356913. 38361923 10.3389/fimmu.2024.1356913 PMC10867145

[29] D. L. Rabenstein , Nat. Prod. Rep. 2002, 19, 312. 12137280 10.1039/b100916h

[30] N. S. Gandhi , C. Freeman , C. R. Parish , R. L. Mancera , Glycobiology 2012, 22, 35. 21746763 10.1093/glycob/cwr095

[31] G. Zhu , H. Song , M. Duan , J. Wang , J. Luo , S. Yang , F. Wu , J. Jiang , J. Chen , W. Tang , Front. Microbiol. 2025, 16, 1559646. 40469724 10.3389/fmicb.2025.1559646 PMC12136495

[32] V. Singh , S. Haque , V. Kumari , H. A. El‐Enshasy , B. N. Mishra , P. Somvanshi , C. K. M. Tripathi , Sci. Rep. 2019, 9, 6482. 31019210 10.1038/s41598-019-42740-7 PMC6482181

[33] K. Ishibashi , H. Iwai , H. Koga , J. Spine Surg. 2019, 5, S115. 31380500 10.21037/jss.2019.04.24 PMC6626749

[34] M. Vital , A. C. Howe , J. M. Tiedje , mBio 2014, 5, 00889. 10.1128/mBio.00889-14 PMC3994512 24757212

[35] R. M. Rani , S. Syngkli , J. Nongkhlaw , B. Das , Biosci. Rep. 2023, 43, BSR20222258. 37021775 10.1042/BSR20222258 PMC10130975

[36] V. Lecomte , N. O. Kaakoush , C. A. Maloney , M. Raipuria , K. D. Huinao , H. M. Mitchell , M. J. Morris , PLoS One 2015, 10, 0126931. 10.1371/journal.pone.0126931 PMC4436290 25992554

[37] J. An , X. Zhao , Y. Wang , J. Noriega , A. T. Gewirtz , J. Zou , PLoS Pathog. 2021, 17, 1009497. 10.1371/journal.ppat.1009497 PMC8049485 33819308

[38] X. Zheng , X. Xu , M. Liu , J. Yang , M. Yuan , C. Sun , Q. Zhou , J. Chen , B. Liu , Fish Shellfish Immunol. 2024, 146, 109376. 38218421 10.1016/j.fsi.2024.109376

[39] W. J. Wang , T. F. Huang , Thromb. Haemost. 2001, 86, 1077. 11686327

[40] Y. Yamazaki , H. Koike , Y. Sugiyama , K. Motoyoshi , T. Wada , S. Hishinuma , M. Mita , T. Morita , Eur. J. Biochem. 2002, 269, 2708. 12047379 10.1046/j.1432-1033.2002.02940.x

[41] T. Deng , D. Gao , X. Song , Z. Zhou , L. Zhou , M. Tao , Z. Jiang , L. Yang , L. Luo , A. Zhou , L. Hu , H. Qin , M. Wu , Nat. Commun. 2023, 14, 396. 36693849 10.1038/s41467-023-35907-4 PMC9873654

[42] Q. Zhu , R. Li , Y. Yan , Y. Huang , H. Su , Y. Dong , X. Pan , W. Sun , X. Wang , H. Meng , R. Wang , H. Ouyang , Y. Hong , Adv. Funct. Mater. 2024, 34, 2402734.

[43] Y. Huang , R. W. Mahley , Neurobiol. Dis. 2014, 72, 3. 25173806 10.1016/j.nbd.2014.08.025 PMC4253862

[44] K. H. Weisgraber , Adv. Protein Chem. 1994, 45, 349. 10.1016/s0065-3233(08)60642-7 8154371

[45] M. F. Lanfranco , C. A. Ng , G. W. Rebeck , Int. J. Mol. Sci. 2020, 21, 6336. 32882843 10.3390/ijms21176336 PMC7503657

[46] A. D. Cardin , N. Hirose , D. T. Blankenship , R. L. Jackson , J. A. Harmony , D. A. Sparrow , J. T. Sparrow , Biochem. Biophys. Res. Commun. 1986, 134, 783. 3947350 10.1016/s0006-291x(86)80489-2

[47] C. Yue , C. Chu , J. Zhao , H. Zhang , W. Chen , Q. Zhai , J. Funct. Foods 2022, 93, 105093.

[48] M. J. Liu , J. Y. Yang , Z. H. Yan , S. Hu , J. Q. Li , Z. X. Xu , Y. P. Jian , Clin. Nutr. 2022, 41, 2333. 36113229 10.1016/j.clnu.2022.08.029

[49] M. Sakamoto , A. Takagaki , K. Matsumoto , Y. Kato , K. Goto , Y. Benno , Int. J. Syst. Evol. Microbiol. 2009, 59, 1748. 19542124 10.1099/ijs.0.007674-0

[50] L. F. Gomez‐Arango , H. L. Barrett , H. D. McIntyre , L. K. Callaway , M. Morrison , D. Nitert , Hypertension 2016, 68, 974. 27528065 10.1161/HYPERTENSIONAHA.116.07910

[51] L. Zhang , C. Liu , Q. Jiang , Y. Yin , Trends Endocrinol. Metab. 2021, 32, 159. 33461886 10.1016/j.tem.2020.12.003

[52] Y. Lei , L. Tang , S. Liu , S. Hu , L. Wu , Y. Liu , M. Yang , S. Huang , X. Tang , T. Tang , X. Zhao , I. Vlodavsky , S. Zeng , B. Tang , S. Yang , Microbiome 2021, 9, 115. 34016163 10.1186/s40168-021-01065-2 PMC8138927

[53] P. S. Rawat , A. S. Seyed Hameed , X. Meng , W. Liu , Gut Microbes 2022, 14, 2068367. 35482895 10.1080/19490976.2022.2068367 PMC9067506

[54] X. Sang , S. Li , R. Guo , Q. Yan , C. Liu , Y. Zhang , Q. Lv , L. Wu , J. Ma , W. You , L. Feng , W. Sun , Front. Microbiol. 2023, 14, 1265425. 37854337 10.3389/fmicb.2023.1265425 PMC10579591

[55] A. Gupta , P. Khanal , J. Agric. Food Res. 2024, 18, 101330.

[56] M. A. Singer , Comp. Biochem. Physiol. B Biochem. Mol. Biol. 2003, 134, 543. 12670782 10.1016/s1096-4959(03)00027-7

[57] G. Stewart , Br. J. Pharmacol. 2011, 164, 1780. 21449978 10.1111/j.1476-5381.2011.01377.x PMC3246703

[58] M. M. Adeva , G. Souto , N. Blanco , C. Donapetry , Metabolism 2012, 61, 1495. 22921946 10.1016/j.metabol.2012.07.007

[59] W. Barmore , F. Azad , W. L. Stone , in Physiology, Urea Cycle, Treasure Island: StatPearls Publishing, Florida 2023. 30020695

[60] M. D. Regan , E. Chiang , Y. Liu , M. Tonelli , K. M. Verdoorn , S. R. Gugel , G. Suen , H. V. Carey , F. M. Assadi‐Porter , Science 2022, 375, 460. 35084962 10.1126/science.abh2950 PMC8936132

[61] D. M. Emms , S. Kelly , Genome Biol. 2015, 16, 157. 26243257 10.1186/s13059-015-0721-2 PMC4531804

[62] D. M. Emms , S. Kelly , Genome Biol. 2019, 20, 238. 31727128 10.1186/s13059-019-1832-y PMC6857279

[63] L. T. Nguyen , H. A. Schmidt , A. von Haeseler , B. Q. Minh , Mol. Biol. Evol. 2015, 32, 268. 25371430 10.1093/molbev/msu300 PMC4271533

[64] Z. Yang , Mol. Biol. Evol. 2007, 24, 1586. 17483113 10.1093/molbev/msm088

[65] J. O. Wertheim , B. Murrell , M. D. Smith , S. L. Kosakovsky Pond , K. Scheffler , Mol. Biol. Evol. 2015, 32, 820. 25540451 10.1093/molbev/msu400 PMC4327161

[66] S. L. Pond , S. D. Frost , S. V. Muse , Bioinformatics 2005, 21, 676. 15509596 10.1093/bioinformatics/bti079

[67] T. De Bie , N. Cristianini , J. P. Demuth , M. W. Hahn , Bioinformatics 2006, 22, 1269. 16543274 10.1093/bioinformatics/btl097

[68] D. Bu , H. Luo , P. Huo , Z. Wang , S. Zhang , Z. He , Y. Wu , L. Zhao , J. Liu , J. Guo , S. Fang , W. Cao , L. Yi , Y. Zhao , L. Kong , Nucleic Acids Res. 2021, 49, W317. 34086934 10.1093/nar/gkab447 PMC8265193

[69] M. Ashburner , C. A. Ball , J. A. Blake , D. Botstein , H. Butler , J. M. Cherry , A. P. Davis , K. Dolinski , S. S. Dwight , J. T. Eppig , M. A. Harris , D. P. Hill , L. Issel‐Tarver , A. Kasarskis , S. Lewis , J. C. Matese , J. E. Richardson , M. Ringwald , G. M. Rubin , G. Sherlock , Nat. Genet. 2000, 25, 25. 10802651 10.1038/75556 PMC3037419

[70] M. Kanehisa , M. Furumichi , Y. Sato , Y. Matsuura , M. K. Ishiguro‐Watanabe , Nucleic Acids Res. 2025, 53, D672. 39417505 10.1093/nar/gkae909 PMC11701520

[71] M. Milacic , D. Beavers , P. Conley , C. Gong , M. Gillespie , J. Griss , R. Haw , B. Jassal , L. Matthews , B. May , R. Petryszak , E. Ragueneau , K. Rothfels , C. Sevilla , V. Shamovsky , R. Stephan , K. Tiwari , T. Varusai , J. Weiser , A. Wright , G. Wu , L. Stein , H. Hermjakob , P. D'Eustachio , Nucleic Acids Res. 2024, 52, D672. 37941124 10.1093/nar/gkad1025 PMC10767911

[72] T. Wu , E. Hu , S. Xu , M. Chen , P. Guo , Z. Dai , T. Feng , L. Zhou , W. Tang , L. Zhan , X. Fu , S. Liu , X. Bo , G. Yu , Innovation 2021, 2, 100141. 34557778 10.1016/j.xinn.2021.100141 PMC8454663

[73] C. Yan , W. Wu , W. Dong , B. Zhu , J.‐T. Li , Innovation 2022, 3, 100295. 36032194 10.1016/j.xinn.2022.100295 PMC9405097

[74] A. Dobin , C. A. Davis , F. Schlesinger , J. Drenkow , C. Zaleski , S. Jha , P. Batut , M. Chaisson , T. R. Gingeras , Bioinformatics 2013, 29, 15. 23104886 10.1093/bioinformatics/bts635 PMC3530905

[75] B. Li , C. N. Dewey , BMC Bioinform. 2011, 12, 323. 10.1186/1471-2105-12-323 PMC3163565 21816040

[76] M. I. Love , W. Huber , S. Anders , Genome Biol. 2014, 15, 550. 25516281 10.1186/s13059-014-0550-8 PMC4302049

[77] M. D. Robinson , D. J. McCarthy , G. K. Smyth , Bioinformatics 2010, 26, 139. 19910308 10.1093/bioinformatics/btp616 PMC2796818

[78] P. Langfelder , S. Horvath , BMC Bioinform. 2008, 9, 559. 10.1186/1471-2105-9-559 PMC2631488 19114008

[79] P. Shannon , A. Markiel , O. Ozier , N. S. Baliga , J. T. Wang , D. Ramage , N. Amin , B. Schwikowski , T. Ideker , Genome Res. 2003, 13, 2498. 14597658 10.1101/gr.1239303 PMC403769

[80] V. Demichev , C. B. Messner , S. I. Vernardis , K. S. Lilley , M. Ralser , Nat. Methods 2020, 17, 41. 31768060 10.1038/s41592-019-0638-x PMC6949130

[81] Y. Choi , G. E. Sims , S. Murphy , J. R. Miller , A. P. Chan , PLoS One 2012, 7, 46688. 10.1371/journal.pone.0046688 PMC3466303 23056405

[82] J. Jumper , R. Evans , A. Pritzel , T. Green , M. Figurnov , O. Ronneberger , K. Tunyasuvunakool , R. Bates , A. Zidek , A. Potapenko , A. Bridgland , C. Meyer , S. A. A. Kohl , A. J. Ballard , A. Cowie , B. Romera‐Paredes , S. Nikolov , R. Jain , J. Adler , T. Back , S. Petersen , D. Reiman , E. Clancy , M. Zielinski , M. Steinegger , M. Pacholska , T. Berghammer , S. Bodenstein , D. Silver , O. Vinyals , et al., Nature 2021, 596, 583. 34265844 10.1038/s41586-021-03819-2 PMC8371605

[83] L. Schrodinger , The PyMOL Molecular Graphics System 2010.

[84] L. Du , C. Geng , Q. Zeng , T. Huang , J. Tang , Y. Chu , K. D. Zhao , Brief Bioinform. 2023, 24, bbad047. 36764832 10.1093/bib/bbad047

[85] J. Abramson , J. Adler , J. Dunger , R. Evans , T. Green , A. Pritzel , O. Ronneberger , L. Willmore , A. J. Ballard , J. Bambrick , S. W. Bodenstein , D. A. Evans , C.‐C. Hung , M. O'Neill , D. Reiman , K. Tunyasuvunakool , Z. Wu , A. Zemgulyte , E. Arvaniti , C. Beattie , O. Bertolli , A. Bridgland , A. Cherepanov , M. Congreve , A. I. Cowen‐Rivers , A. Cowie , M. Figurnov , F. B. Fuchs , H. Gladman , R. Jain , et al., Nature 2024, 630, 493. 38718835 10.1038/s41586-024-07487-w PMC11168924

[86] G. V. Uritskiy , J. DiRuggiero , J. Taylor , Microbiome 2018, 6, 158. 30219103 10.1186/s40168-018-0541-1 PMC6138922

[87] J. Lu , F. P. Breitwieser , P. Thielen , S. L. Salzberg , PeerJ. Comput. Sci. 2017, 3, 104. 10.7717/peerj-cs.104 PMC12016282 40271438

[88] Y. X. Liu , Y. Qin , T. Chen , M. Lu , X. Qian , X. Guo , Y. Bai , Protein Cell 2021, 12, 315. 32394199 10.1007/s13238-020-00724-8 PMC8106563

[89] A. Blanco‐Míguez , F. Beghini , F. Cumbo , L. J. McIver , K. N. Thompson , M. Zolfo , P. Manghi , L. Dubois , K. D. Huang , A. M. Thomas , W. A. Nickols , G. Piccinno , E. Piperni , M. Puncochr , M. Valles‐Colomer , A. Tett , F. Giordano , R. Davies , J. Wolf , S. E. Berry , T. D. Spector , E. A. Franzosa , E. Pasolli , F. Asnicar , C. Huttenhower , N. Segata , Nat. Biotechnol. 2023, 41, 1633. 36823356 10.1038/s41587-023-01688-w PMC10635831

[90] M. Shaffer , M. A. Borton , B. B. McGivern , A. A. Zayed , S. L. La Rosa , L. M. Solden , P. Liu , A. B. Narrowe , J. Rodríguez‐Ramos , B. Bolduc , M. C. Gazitúa , R. A. Daly , G. J. Smith , D. R. Vik , P. B. Pope , M. B. Sullivan , S. Roux , K. C. Wrighton , Nucleic Acids Res. 2020, 48, 8883. 32766782 10.1093/nar/gkaa621 PMC7498326

