# Supplementary material for: Hologenomics Reveals Specialized Dietary Adaptations in the Mengla Snail‐Eating Snake
Source: Adv Sci (Weinh). 2025 Jul 16;12(40):e09999. doi: 10.1002/advs.202509999 (PMC12561409; doi:10.1002/advs.202509999)
Supplement: Supplementary file 1 — Supporting Information [file ADVS-12-e09999-s001.docx]

Supporting Information

**Hologenomics Reveals Specialized Dietary Adaptations in the Mengla Snail-eating Snake**

*Chaochao Yan, Xin-Ning Li, Zhong-Liang Peng, Wei Wu, Zeng Wang, Zhao-Ran Zhu, Jia-Chang Liu, Yao Wang, Jin-Long Ren, Zhi-Yi Zhang^*^, Jia-Tang Li^*^*

# **Supplementary tables**

## Table S1. Genomes of 31 species used in the comparative genomic analysis.

| ID | Family | Scientifc Name | Common Name | Accession NO./websit |
| --- | --- | --- | --- | --- |
| Pgut | Colubridae | *Pantherophis guttatus* | Red cornsnake | NCBI: GCF_001185365.1* |
| Tbai | Dipsadidae | *Thermophis baileyi* | Xizang hot-spring snake | <https://ngdc.cncb.ac.cn>, PRJCA007342 |
| Pbiv | Pythonidae | *Python bivittatus* | Burmese python | NCBI: GCF_000186305.1* |
| Nnaj | Elapidae | *Naja naja* | Indian cobra | NCBI: GCA_009733165.1* |
| Nscu | Elapidae | *Notechis scutatus* | Mainland tiger snake | NCBI: GCF_900518725.1* |
| Ptex | Elapidae | *Pseudonaja textilis* | Eastern brown snake | NCBI: GCA_900608585.1* |
| Ohan | Elapidae | *Ophiophagus hannah* | King Cobra | NCBI: GCA_000516915.1* |
| Hcur | Elapidae | *Hydrophis curtus* | Shaw's sea snake | https://doi.org/10.6084/m9.figshare.11391606.v5 |
| Bcon | Boidae | *Boa constrictor* | Boa Constrictor | <https://doi.org/10.6084/m9.figshare.9793013.v2> |
| Dacu | Viperidae | *Deinagkistrodon acutus* | Five-pacer viper | ftp.cngb.org/pub/gigadb/pub/10.5524/100001_101000/100196/ |
| Cvir | Viperidae | *Crotalus viridis* | Western Rattlesnake | NCBI: GCA_003400415.2* |
| Cruf | Cylindrophiidae | *Cylindrophis ruffus* | Red-tailed pipe snake | National Genomic Data Center (https://  bigd.big.ac.cn/gwh/):  GWHBWDM00000000. |
| Etat | Erycidae | *Eryx tataricus* | Tartar sand boa | National Genomic Data Center (https://  bigd.big.ac.cn/gwh/):  GWHBWDN00000000. |
| Ajin | Xenodermatidae | *Achalinus jinggangensis* | Zong's odd-scaled snake | National Genomic Data Center (https://  bigd.big.ac.cn/gwh/):  GWHBWDV00000000. |
| Pmen | Pareatidae | *Pareas menglaensis* | Keeled slug snake | National Genomic Data Center (https://  bigd.big.ac.cn/gwh/):  GWHBWDS00000000. |
| Gshe | Viperidae | *Gloydius shedaoensis* | Shedao island pitviper | National Genomic Data Center (https://  bigd.big.ac.cn/gwh/):  GWHBWDU00000000. |
| Hplu | Homalopsidae | *Hypsiscopus plumbea* | Plumbeous water snake | National Genomic Data Center (https://  bigd.big.ac.cn/gwh/):  GWHBWDO00000000. |
| Bful | Lamprophiidae | *Boaedon fuliginosus* | African house snake | National Genomic Data Center (https://  bigd.big.ac.cn/gwh/):  GWHBWDQ00000000. |
| Ppul | Lamprophiidae | *Psammodynastes pulverulentus* | Common Mock Viper | National Genomic Data Center (https://  bigd.big.ac.cn/gwh/):  GWHBWDP00000000 |
| Csep | Calamariidae | *Calamaria septentrionalis* | Hong kong dwarf snake | National Genomic Data Center (https://  bigd.big.ac.cn/gwh/):  GWHBWDL00000000. |
| Apra | Colubridae | *Ahaetulla prasina* | Asian vine snake | NCBI: ASM2864084v1 |
| Asin | Alligatoridae | *Alligator sinensis* | Chinese alligator | NCBI: GCF_000455745.1* |
| Cpic | Emydidae | *Chrysemys picta bellii* | Western Painted Turtle | [ftp.ensembl.org:/pub/release-102/fasta/chrysemys_picta_bellii](ftp://ftp.ensembl.org/pub/release-102/fasta/chrysemys_picta_bellii) |
| Smer | Teiidae | *Salvator merianae* | Argentine black and white tegu | NCBI: GCA_003586115.2* |
| Pmur | Lacertidae | *Podarcis muralis* | common wall lizard | NCBI: GCF_004329235.1* |
| Vkom | Varanidae | *Varanus komodoensis* | The Komodo dragon | NCBI: GCF_004798865.1* |
| Eper | Colubridae | *Euprepiophis perlacea* | Pearl-banded Rat Snake | National Genomic Data Center (https://  bigd.big.ac.cn/gwh/):  GWHBOZS00000000. |
| Xhai | Xenopeltidae | *Xenopeltis hainanensis* | Hainan Sunbeam Snake | National Genomic Data Center([https://bigd.big.ac.cn/gwh/](https://bigd.big.ac.cn/gwh/)):  GWHBWDW00000000. |
| Oapo | Anguidae | *Ophisaurus apodus* | European Glass Lizard | China National GeneBank Nucleotide Sequence Archive (https://www.cngb.org) :  CNP0003553 |
| Lnat | Leptotyphlopidae | *Leptotyphlops natatrix* | Gambia Blind Snake | National Genomic Data Center ([https://bigd.big.ac.cn/gwh/](https://bigd.big.ac.cn/gwh/)): GWHBWDR00000000. |
| Tdia | Typhlopidae | *Typhlops diardii* | Diard's blind snake | National Genomic Data Center (https://  bigd.big.ac.cn/gwh/):  GWHBWDT00000000. |

Table S2. Sepecies-specific expanded Gene Familes for *Pareas menglaensis.*

## Table S3. KEGG and GO enrichment of expanded Gene Familes in *Pareas menglaensis.*

Table S4. Contracted gene families in *Pareas menglaensis* related to *Pantherophis guttatus.*

## Table S5 . Rapidly evolving genes (1846) estimated by branch model in PAML with *Pareas menglaensis* setting as foreground.

## Table S6. Positively Selected Genes (963) estimated by site-branch model in PAML with *Pareas menglaensis* setting as foreground.

## Table S7. KEGG and GO enrichment of rapidly evolving genes (1846) estimated by branch model in PAML with *Pareas menglaensis* setting as foreground.

## Table S8. KEGG and GO enrichment of positively selected genes (963) estimated by site-branch model in PAML with *Pareas menglaensis* setting as foreground.

Table S9. Positively Selected Genes identified by HYPHY FEL program between Pmen and other species.

Table S10. Rapidly Evolved Genes identified by HYPHY RELAX program between Pmen and other species.

Table S11. KEGG and GO enrichment of positively selected genes identified by HYPHY FEL program between Pmen and other species.

Table S12. KEGG and GO enrichment of rapidly evolved genes identified by HYPHY RELAX program between Pmen and other species.

Table S13. KEGG and GO enrichment of genes with *Pareas menglaensis*-specific mutations.

Table S14. Overlaps between different types of genes.

## Table S15. Docking parameters of the best pose between APOE and ligand (Cholesterol) for *Pareas menglaensis* APOE and non-*Pareas* *menglaensis* APOE.

| Receptor | *Pareas menglaensis* APOE | Non-*Pareas menglaensis* APOE |
| --- | --- | --- |
| Ligand | Cholesterol | Cholesterol |
| Energy | -7.61 | -6.79 |
| cRMSD | 0 | 0 |
| rRMSD | 10.73 | 17.06 |
| logKi | -5.58 | -4.976 |
| LE | 0.272 | 0.242 |
| SILE | 2.801 | 2.499 |
| FQ | 0.762 | 0.678 |
| LLE | -1.809 | -2.413 |
| LELP | 27.164 | 30.532 |
| Ki | 2.63 uM | 10.56 uM |

## TableS16. Differently expressed genes between infralabial gland and other tissues in *Pareas menglaensis.*

## TableS17. KEGG and GO enrichment of differently expressed genes in the infralabial gland of *Pareas menglaensis* compared to other tissues.

## TableS18. Differently expressed genes of infralabial gland of *Pareas menglaensis* between fasting and post-meal states (reference).

## TableS19. Differently expressed protein of infralabial gland of *Pareas menglaensis* between fasting (reference) and post-meal states.

## TableS20. KEGG and GO enrichment of differently expressed protein of infralabial gland of *Pareas menglaensis* between fasting and post-meal states.

## TableS21. KEGG and GO enrichment of differently expressed genes of infralabial gland of *Pareas menglaensis* between Fasting and post-meal states.

## Table S22. Infralabial gland associated gene module (steelblue) identified by WGCNA.

## Table S23. KEGG and GO enrichment of genes in steelblue module that is significantly associated with infralabial gland of *Pareas menglaensis* compared to other tissues.

## Table S24. Alpha diversities measures for *Pareas menglaensis* and other snakes used in metagenome comparison.

| SampleID | Species | Berger-parker | shannon | simpson | invsimpson |
| --- | --- | --- | --- | --- | --- |
| LJT-BN2023167d | *Pareas menglaensis* | 0.958231004 | 4.287541207 | 0.958231004 | 23.941202709 |
| LJT-BN2023168d | *Pareas menglaensis* | 0.960498382 | 4.492397569 | 0.960498382 | 25.315418596 |
| LJT-BN2023169d | *Pareas menglaensis* | 0.957088613 | 4.643111665 | 0.957088613 | 23.303837518 |
| LJT-BN2023170d | *Pareas menglaensis* | 0.952049271 | 4.279266220 | 0.952049271 | 20.854740195 |
| LJT-BN2023171d | *Pareas menglaensis* | 0.756705947 | 2.607753007 | 0.756705947 | 4.110252551 |
| LJT-BN2023172d | *Pareas menglaensis* | 0.961398659 | 4.630899792 | 0.961398660 | 25.905836138 |
| LJT-LAB2024344ILDM | *Naja atra* | 0.854950216 | 3.583616933 | 0.854950216 | 6.894184705 |
| LJT-LAB2024344ILPM | *Naja atra* | 0.817810296 | 3.069338096 | 0.817810296 | 5.488784379 |
| LJT-LAB2024346ILPM | *Naja atra* | 0.840602882 | 3.167422834 | 0.840602882 | 6.273639167 |
| LJT-LAB2024350ILM | *Naja atra* | 0.920499709 | 4.326928152 | 0.920499708 | 12.578570250 |
| LJT-LAB2024351ILM | *Naja atra* | 0.942831954 | 5.177164528 | 0.942831954 | 17.492289237 |
| LJT-LAB2024352ILM | *Naja atra* | 0.857964110 | 3.248846114 | 0.857964110 | 7.040474075 |
| Ozh24019Imic | *Opisthotropis zhaoermii* | 0.650352679 | 1.868845611 | 0.650352679 | 2.860024775 |
| Ozh24027Imic | *Opisthotropis zhaoermii* | 0.709754629 | 2.805920415 | 0.709754629 | 3.445360718 |
| Ozh24032Imic | *Opisthotropis zhaoermii* | 0.676547605 | 2.346375456 | 0.538835128 | 2.168421883 |

## Table S25. Significantly differently enriched biological functions of intestinal flora between *Pareas menglaensis* and other snakes.

## Table S26. Significantly different abundance of microbial genes between *Pareas menglaensis* and others.

## Table S27. Tissues of samples used for different type of sequencing.

## Table S28. Sample information of *Pareas menglaensis* used in sequencing.

| sampleID | species | place | dealing |
| --- | --- | --- | --- |
| LJT-BN2023168 | *Pareas menglaensis* | Xishuangbanna, Yunnan | fasting |
| LJT-BN2023169 | *Pareas menglaensis* | Xishuangbanna, Yunnan | fasting |
| LJT-BN2023170 | *Pareas menglaensis* | Xishuangbanna, Yunnan | fasting |
| LJT-BN2023237 | *Pareas menglaensis* | Xishuangbanna, Yunnan | meal |
| LJT-BN2023238 | *Pareas menglaensis* | Xishuangbanna, Yunnan | meal |
| LJT-BN2023239 | *Pareas menglaensis* | Xishuangbanna, Yunnan | meal |
| LJT-BN2023242 | *Pareas menglaensis* | Xishuangbanna, Yunnan | meal |

## Table S29. Sample information used in metagenome sequencing.

| sampleID | host | sampling site | geographic_location |
| --- | --- | --- | --- |
| LJT-BN2023167d | *Pareas menglaensis* | Xishuangbanna, Yunnan | 21.83107° N, 101.37369° E |
| LJT-BN2023168d | *Pareas menglaensis* | Xishuangbanna, Yunnan | 21.829450° N, 101.37284° E |
| LJT-BN2023169d | *Pareas menglaensis* | Xishuangbanna, Yunnan | 21.829450° N, 101.37284° E |
| LJT-BN2023170d | *Pareas menglaensis* | Xishuangbanna, Yunnan | 21.82649° N, 101.37300° E |
| LJT-BN2023171d | *Pareas menglaensis* | Xishuangbanna, Yunnan | 21.81092° N, 101.38296° E |
| LJT-BN2023172d | *Pareas menglaensis* | Xishuangbanna, Yunnan | 21.98253° N, 101.12232° E |
| LJT-LAB2024352 ILM | *Naja atra* | Sichuan (donated) | NA |
| LJT-LAB2024351 ILM | *Naja atra* | Sichuan (donated) | NA |
| LJT-LAB2024350 ILM | *Naja atra* | Sichuan (donated) | NA |
| LJT-LAB2024346 ILPM | *Naja atra* | Sichuan (donated) | NA |
| LJT-LAB2024344 ILPM | *Naja atra* | Sichuan (donated) | NA |
| LJT-LAB2024344 ILDM | *Naja atra* | Sichuan (donated) | NA |
| Ozh24032Imic | *Opisthotropis zhaoermii* | Leishan, Guizhou | 26.37969° N, 108.10049° E |
| Ozh24027Imic | *Opisthotropis zhaoermii* | Leishan, Guizhou | 26.37969° N, 108.10049° E |
| Ozh24019Imic | *Opisthotropis zhaoermii* | Leishan, Chengdu | 26.37969° N, 108.10049° E |

## Table S30. PCR primers used for the acquisition of recombinant HPSEs.

# ****Supplementary figures****


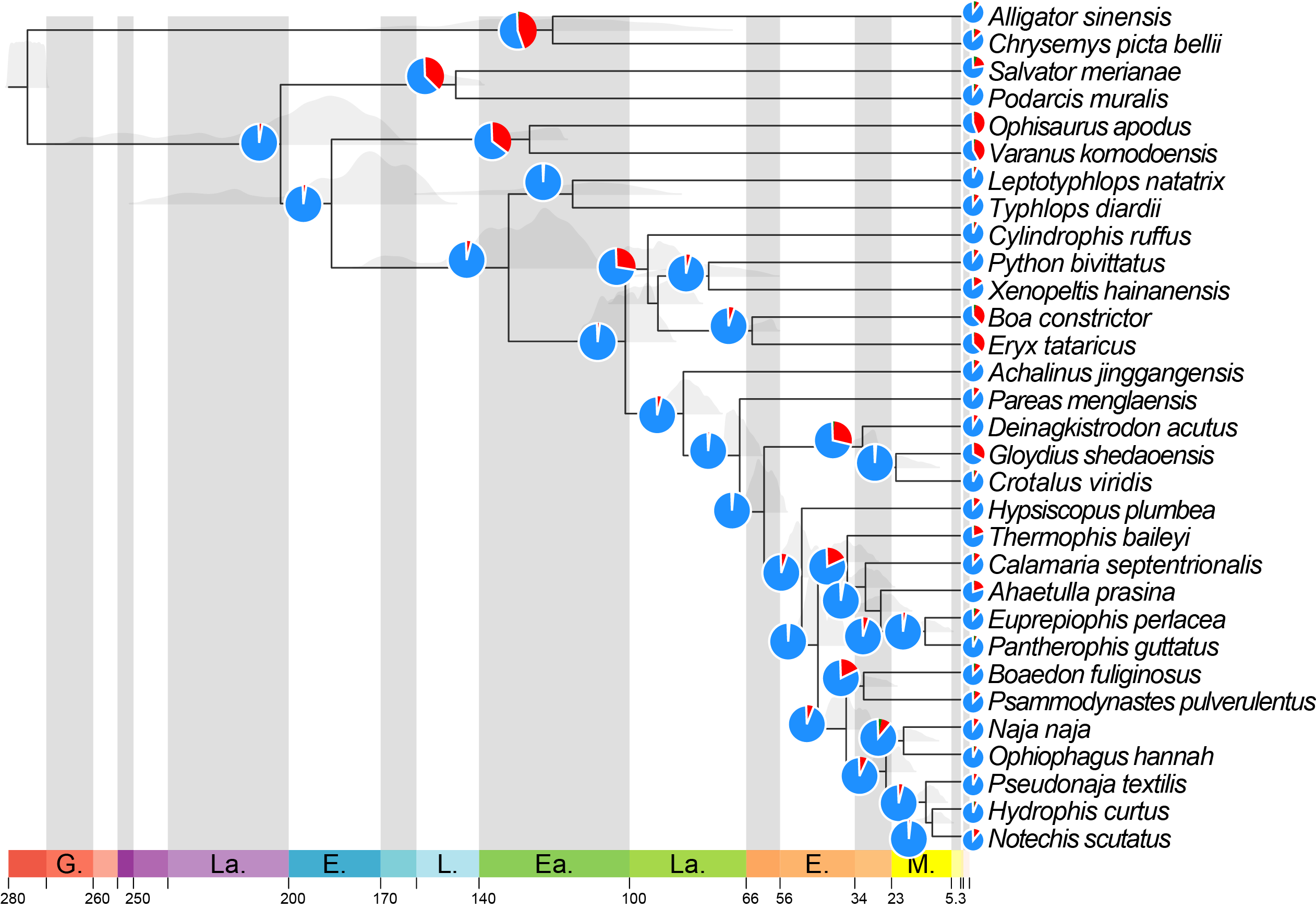


## Figure S1. Phylogenetic Tree of 31 Species Reconstructed Using Single-Copy Orthologous Groups Identified in Genomes. The phylogenetic tree is scaled by divergence time, with geological periods indicated below and divergence times in millions of years ago (Mya) show above. Blue and red numbers denote the number of significantly expanded (red) and contracted (blue) gene families, respectively. Geological time periods are abbreviated as follows: G.: Paleozoic Era, La.: Lower Paleozoic, E.: Mesozoic Era, L.: Lower Mesozoic, Ea.: Early Mesozoic, La.: Late Mesozoic, M.: Cenozoic Era.


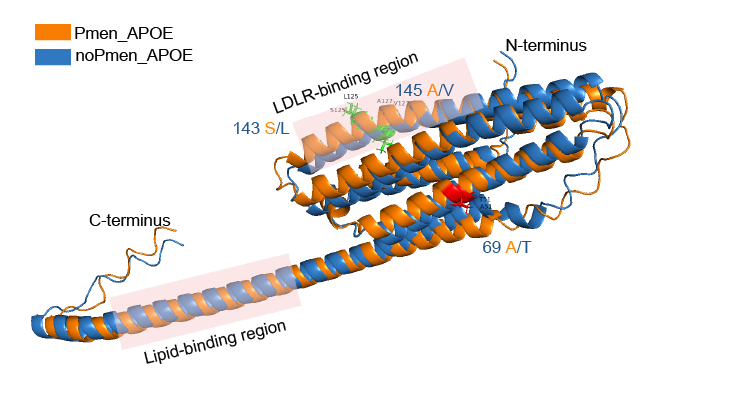


## Figure S2. 3D structure of APOE containing the *Pareas menglaensis*-specific variation L143S and V145A, superimposed with wild-type APOE from *Pareas menglaensis*. The 3D structure, primarily composed of α-helices, were predicted using AlphaFold2. The mutation sites are shown as stick models.


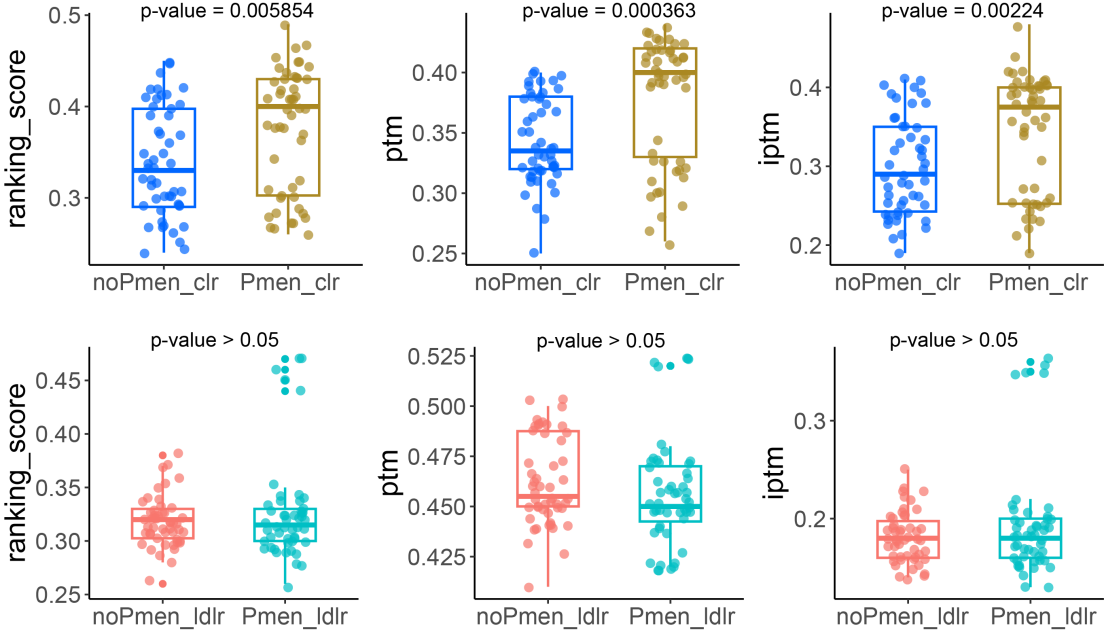


Figure S3. Comparative boxplots of predicted APOE binding properties between *P. menglaensis* (Pmen) and other snakes (noPmen). (Top) APOE-cholesterol interactions demonstrated significant differences (all *p* < 0.01). (Bottom) Simulation of APOE-LDLR interactions showed no systematic divergence (all *p* > 0.05). ptm, predicted template modelling; iptm, interface predicted TM-score. AlphaFold3 simulated binding interactions 50 times. A two-sided Student’s *t*-test was used to assess significant differences. The whiskers indicate 1.5 times of the interquartile range.


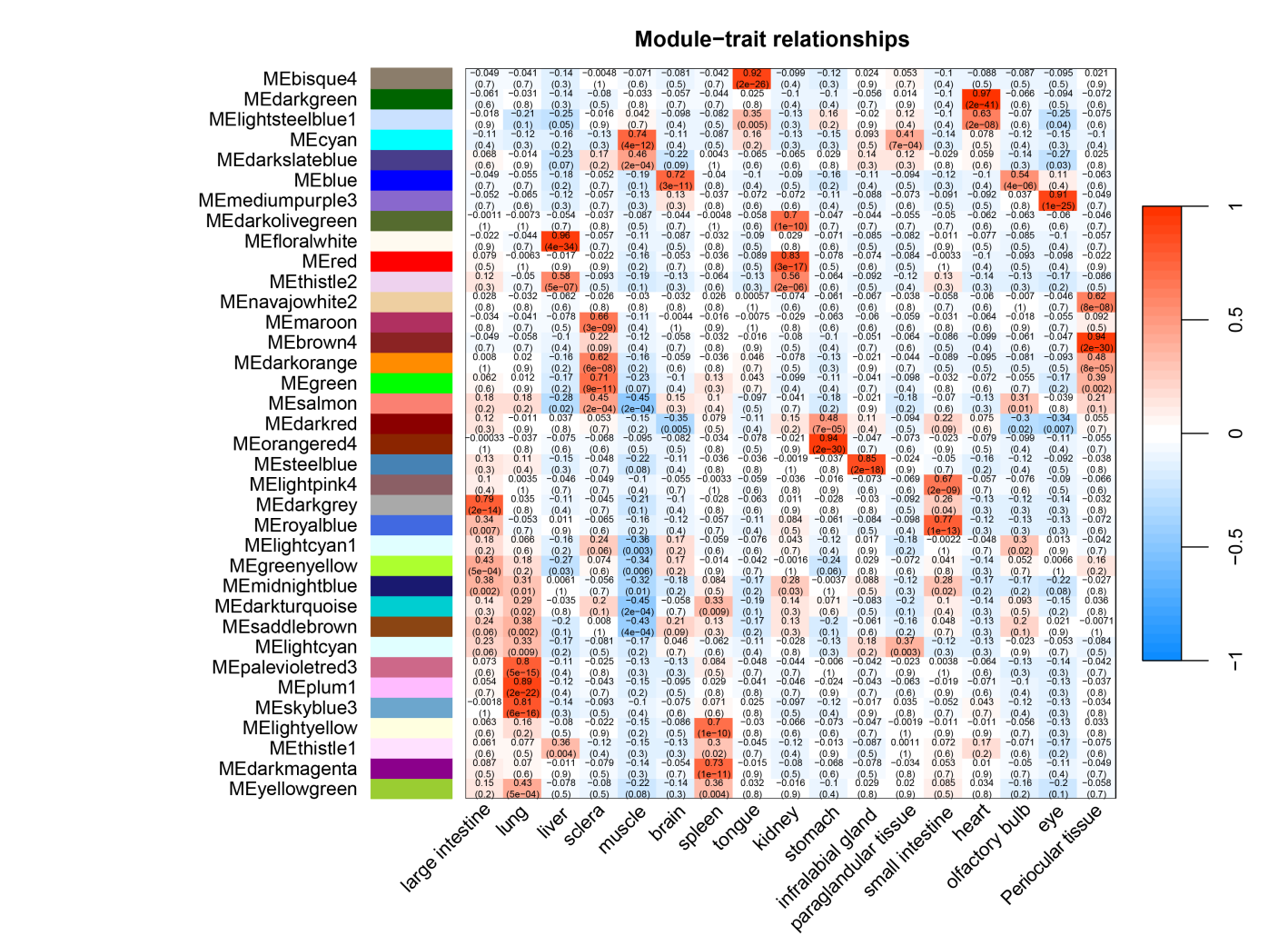


Figure S4. Heatmap of Module-Trait Relationships Produced by WGCNA. The steelblue module, which includes *HPSE*, was significantly associated with the infralabial gland.


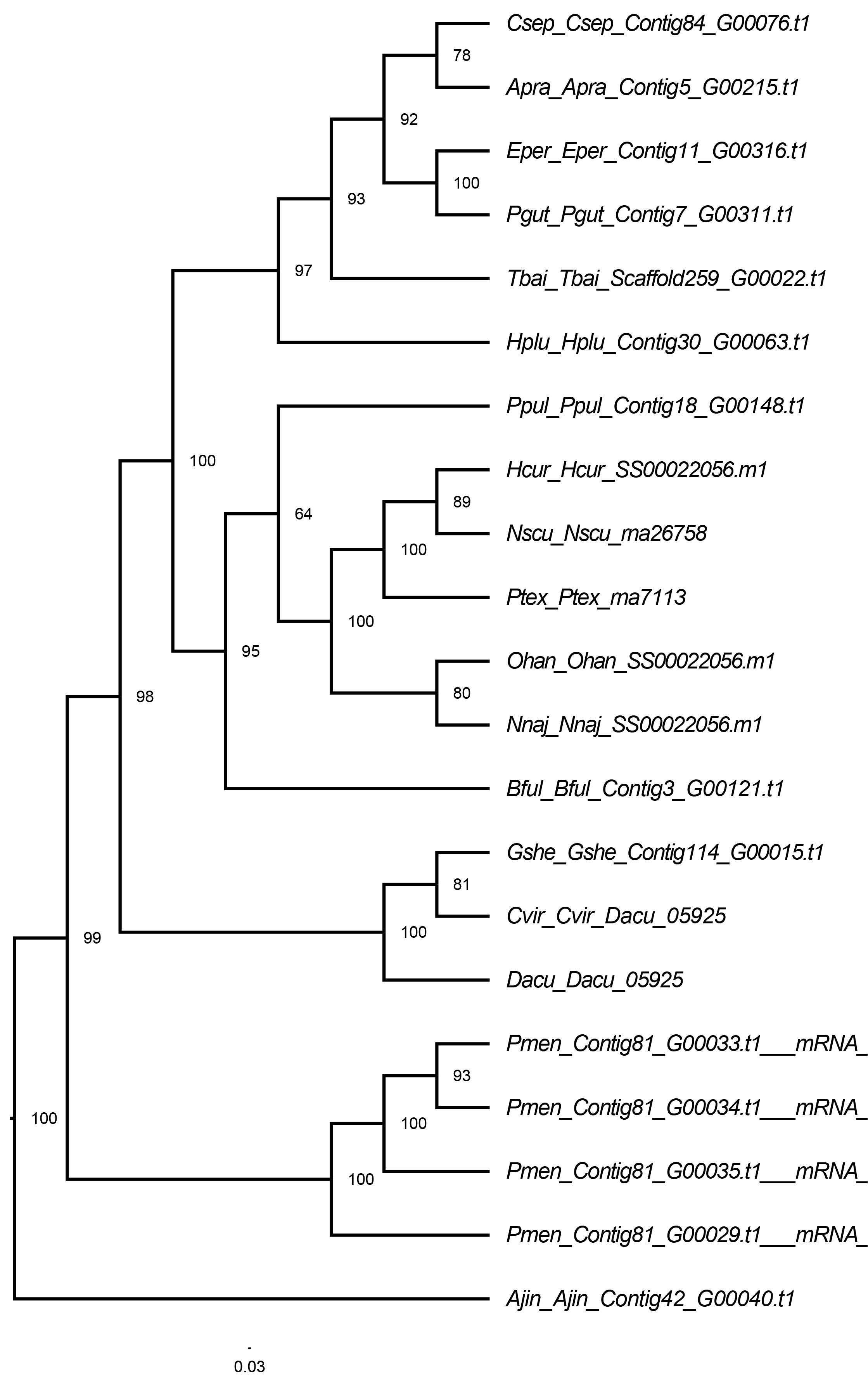


## Figure S5. Phylogenetic Relationships Among *HPSE* Gene Family in Snakes. *Pareas menglaensis* contains four paralogous *HPSE* genes that cluster together, suggesting these genes were duplicated within *Pareas menglaensis* after its divergence from other snakes.


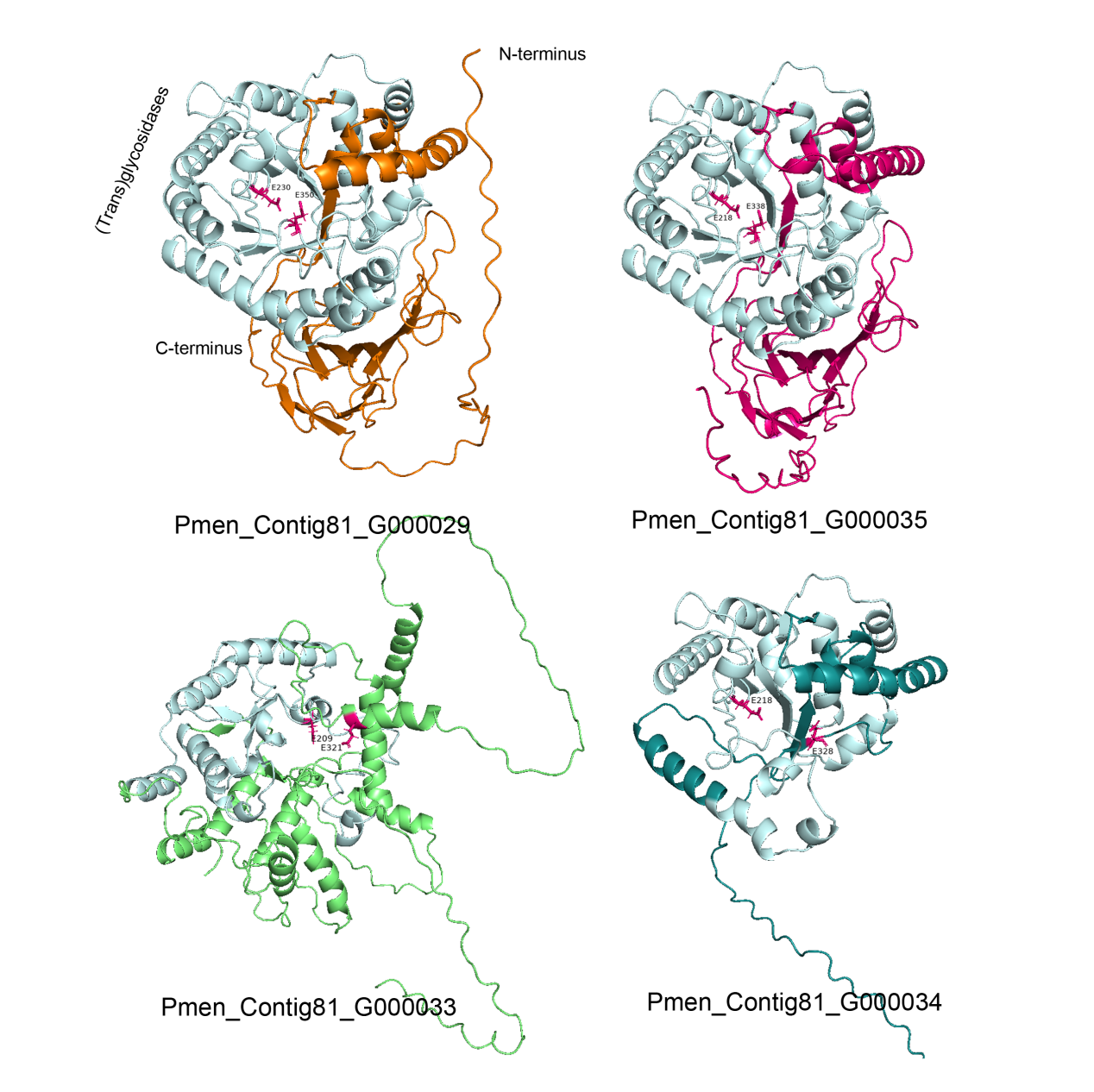


Figure S6. 3D Structures of HPSE Proteins in *Pareas menglaensis*. The structures include HPSE-29 (Pmen_Contig81_G000029), HPSE-35 (Pmen_Contig81_G000035), HPSE-33 (Pmen_Contig81_G000033), and HPSE-34 (Pmen_Contig81_G000034). HPSE-29 and HPSEL-35 are integrated and show similarities when compared to these proteins in other snakes. The functional (Trans) glycosidase regions are shown in pale cyan, while the calculated enzymatic sites are depicted in hot pink as stick models.


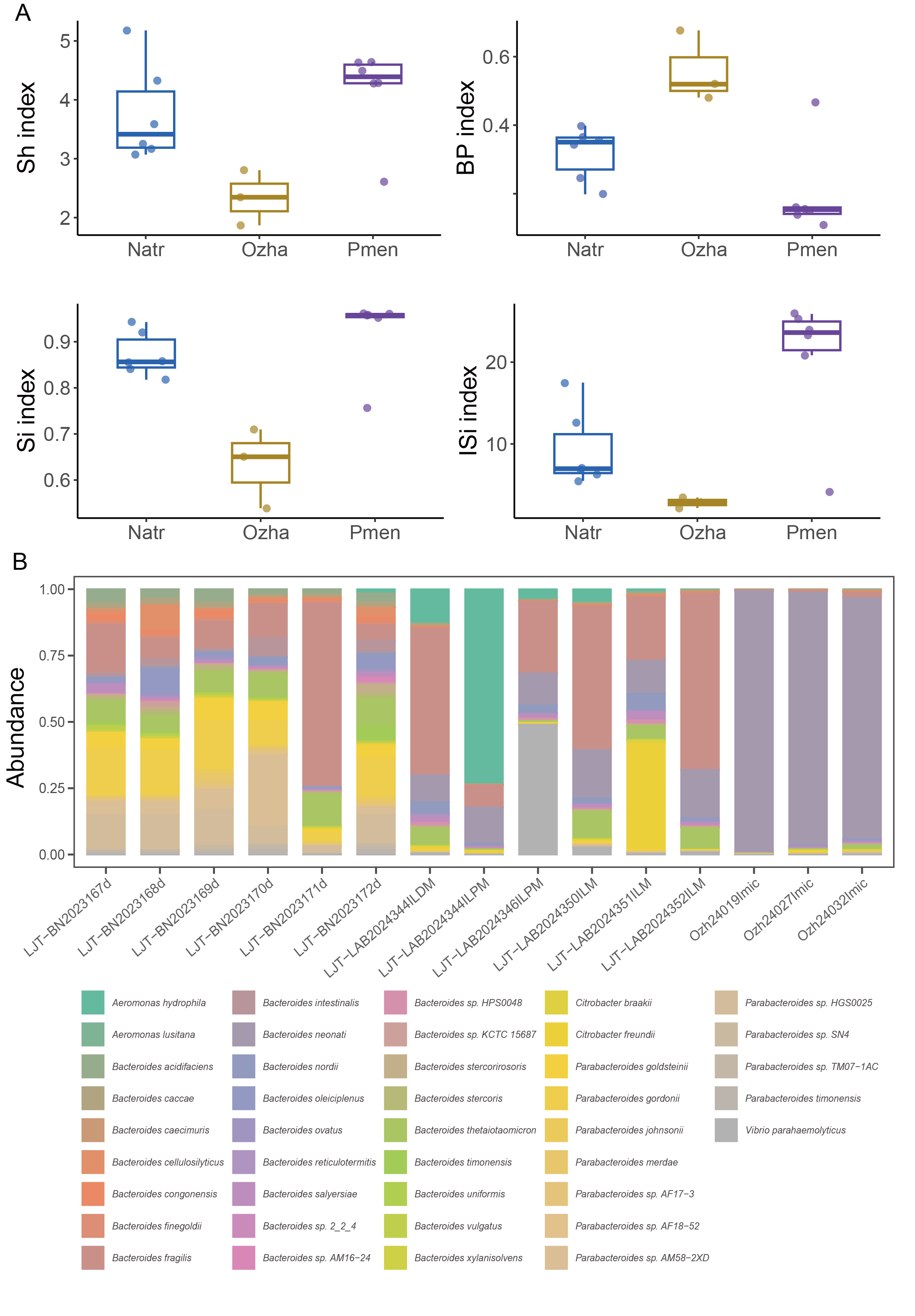


Figure S7. Comparison of gut microbiota diversity and composition across individuals. (A) Boxplots comparing microbial community diversity indices among six *Naja atra* (Natr), three *Opisthotropis zhaoermii* (Ozha), and six *Pareas menglaensis* (Pmen). Sh, Shannon; BP, Berger-parker; Si, Simpson; ISi, Inverse Simpson. The whiskers indicate 1.5 times of the interquartile range. (B) Stacked bar chart displaying the relative abundances of the top 10 dominant microbial taxa in individual samples.


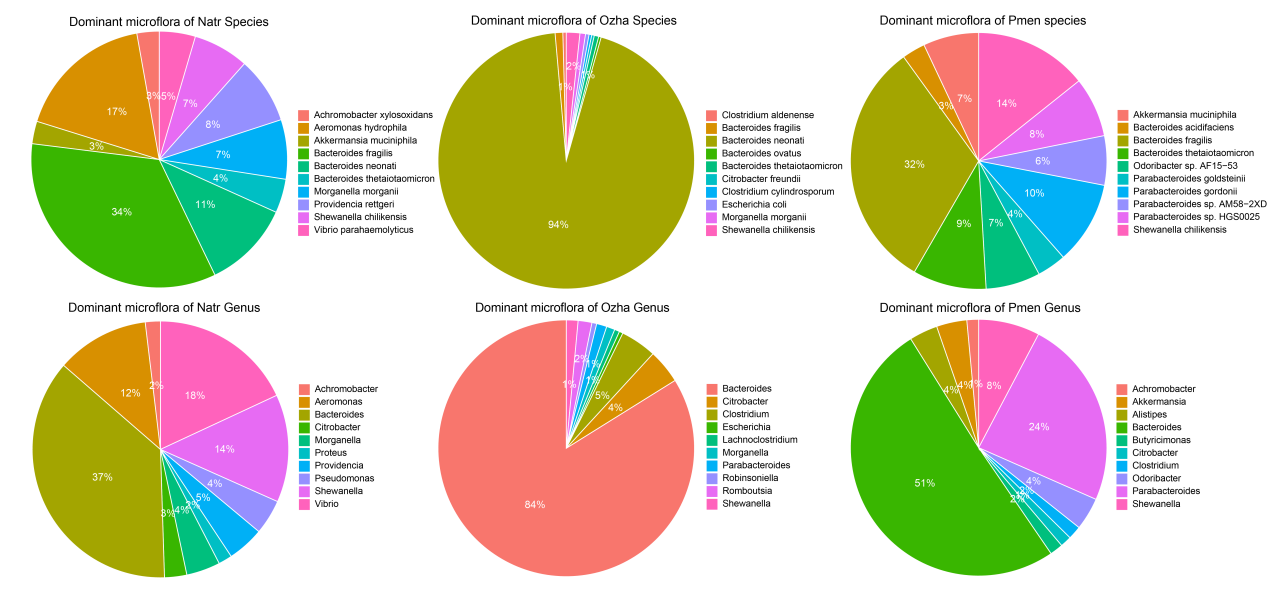


Figure S8. Comparative analysis of gut microbiota composition across *Naja atra* (Natr), *Opisthotropis zhaoermii* (Ozha) *and Pareas menglaensis*(Pmen). (Top) Composition of the top 10 dominant gut microbiota at species level. (Bottom) Composition of the top 10 dominant gut microbiota at genus level.


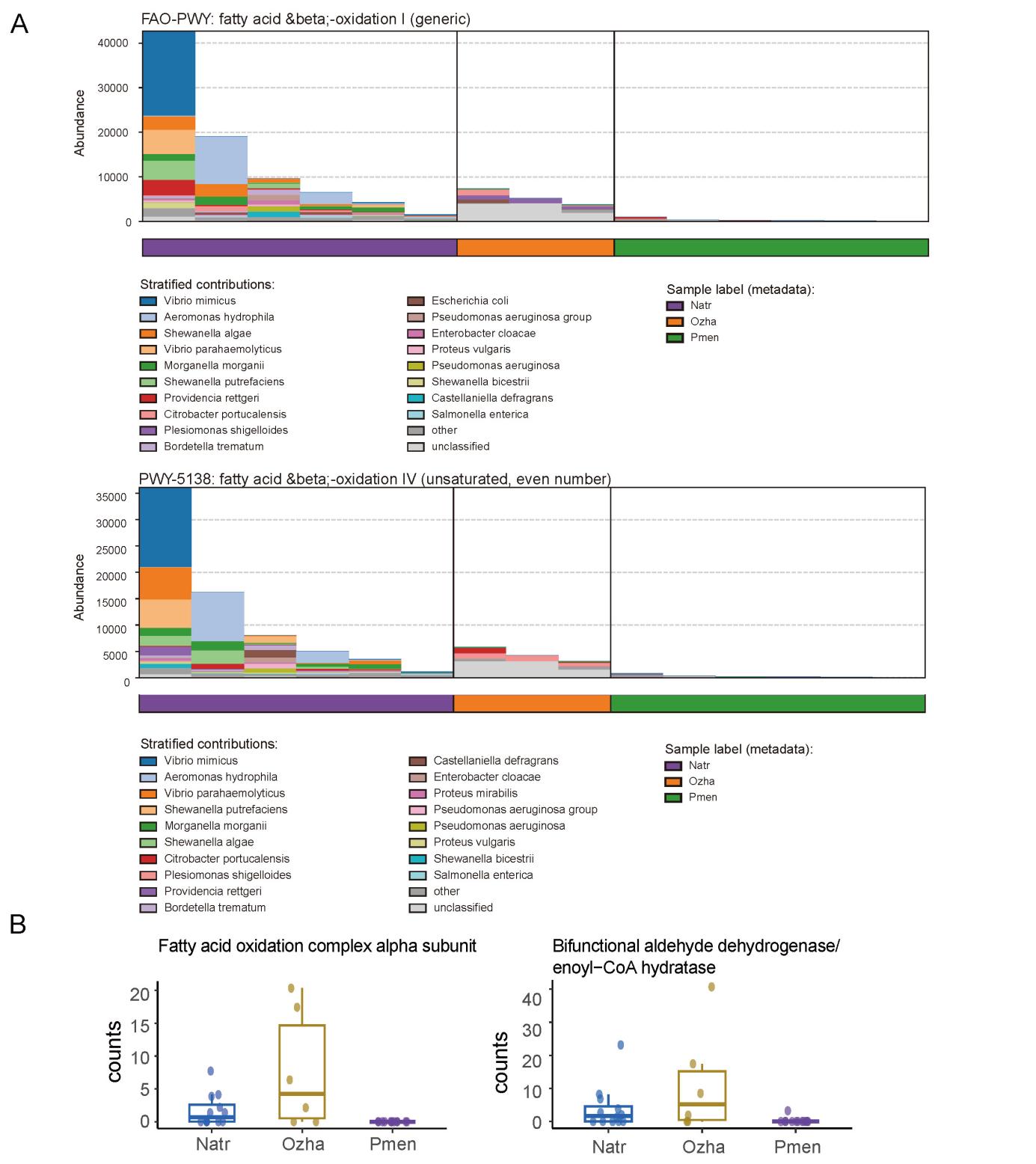


## Figure S9. Functional Enrichment and gene abundance of different Microorganisms between *Pareas menglaensis* and other snakes. (A) The bar graph represents microbial pathways significantly (*p* < 0.05) enriched in gut symbionts with higher abundance in non-snail eating snakes (Natr, Ozha) than in *P. menglaensis* (Pmen). The y-axis quantifies abundance of microbes in *P. menglaensis* (green box) and two other snakes (orange and purple boxes). Colors within each bar denote contributions of different microbes (B) Bar charts showing significant (adjusted *p* < 0.05) differences in abundance of microbial genes, associated with fat breakdown, between Mengla snail-eating snakes and other snakes. The whiskers indicate 1.5 times of the interquartile range.


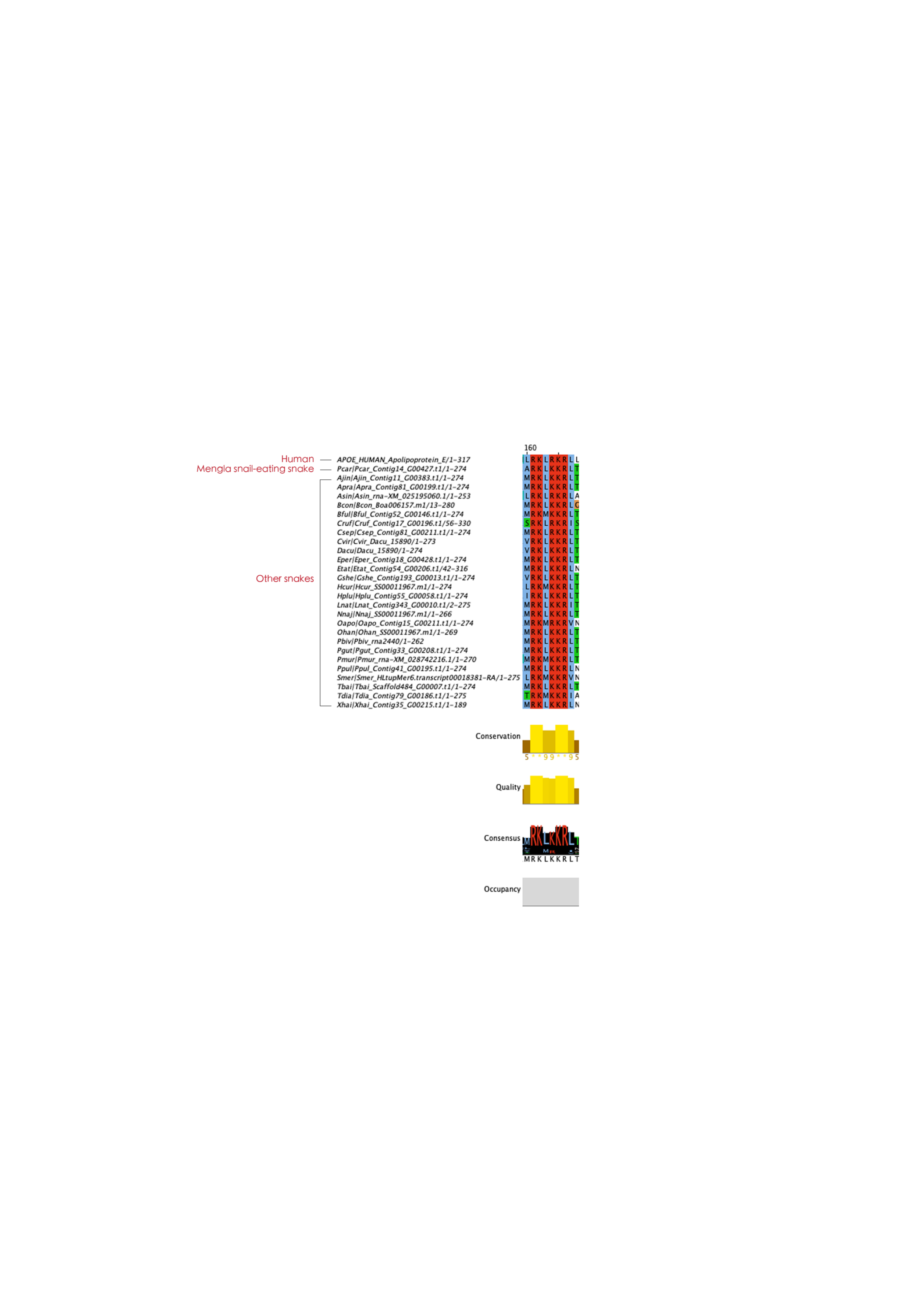


Figure S10. Alignment of APOEs. Alignment of the human APOE sequence with those of snake species used in our study, showing conserved Lys/Arg residues crucial for glycosaminoglycan binding.


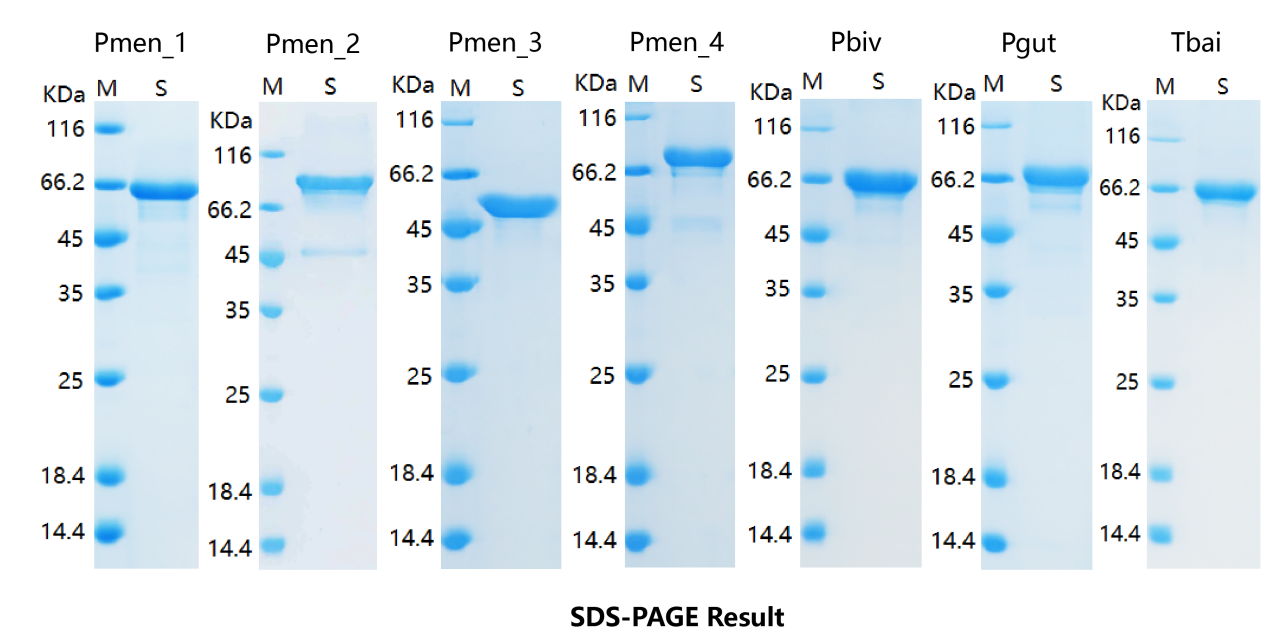


## Figure S11. Gel electrophoresis of prokaryotic expression products of seven recombinant HPSEs. Lane M, protein marker; Lane S, final sample.
